# Branched chain amino acids exacerbate myocardial ischemia/reperfusion vulnerability via enhancing GCN2/ATF6/PPAR-α pathway-dependent fatty acid oxidation

**DOI:** 10.7150/thno.44836

**Published:** 2020-04-27

**Authors:** Yueyang Li, Zhenyu Xiong, Wenjun Yan, Erhe Gao, Hexiang Cheng, Guiling Wu, Yi Liu, Ling Zhang, Congye Li, Shan Wang, Miaomiao Fan, Huishou Zhao, Fuyang Zhang, Ling Tao

**Affiliations:** 1 Department of Cardiology, Xijing Hospital, Air Force Medical University, Xi'an, China.; 2 Department of Cardiology, Tangdu Hospital, Air Force Medical University, Xi'an, China.; 3 Center for Translational Medicine, Temple University, Philadelphia, USA.; 4 School of Aerospace Medicine, Air Force Medical University, Xi'an, China.; 5 Department of Physiology and Pathophysiology, School of Basic Medicine, Air Force Medical University, Xi'an, China.

**Keywords:** Branched chain amino acids, Fatty acid metabolism, Ischemia/reperfusion injury, Peroxisome proliferation-activated receptor-α, Vulnerability.

## Abstract

**Rationale**: Myocardial vulnerability to ischemia/reperfusion (I/R) injury is strictly regulated by energy substrate metabolism. Branched chain amino acids (BCAA), consisting of valine, leucine and isoleucine, are a group of essential amino acids that are highly oxidized in the heart. Elevated levels of BCAA have been implicated in the development of cardiovascular diseases; however, the role of BCAA in I/R process is not fully understood. The present study aims to determine how BCAA influence myocardial energy substrate metabolism and to further clarify the pathophysiological significance during cardiac I/R injury.

**Methods**: Parameters of glucose and fatty acid metabolism were measured by seahorse metabolic flux analyzer in adult mouse cardiac myocytes with or without BCAA incubation**.** Chronic accumulation of BCAA was induced in mice receiving oral BCAA administration. A genetic mouse model with defective BCAA catabolism was also utilized. Mice were subjected to MI/R and the injury was assessed extensively at the whole-heart, cardiomyocyte, and molecular levels.

**Results**: We confirmed that chronic accumulation of BCAA enhanced glycolysis and fatty acid oxidation (FAO) but suppressed glucose oxidation in adult mouse ventricular cardiomyocytes. Oral gavage of BCAA enhanced FAO in cardiac tissues, exacerbated lipid peroxidation toxicity and worsened myocardial vulnerability to I/R injury. Etomoxir, a specific inhibitor of FAO, rescued the deleterious effects of BCAA on I/R injury. Mechanistically, valine, leucine and their corresponding branched chain α-keto acid (BCKA) derivatives, but not isoleucine and its BCKA derivative, transcriptionally upregulated peroxisome proliferation-activated receptor alpha (PPAR-α). BCAA/BCKA induced PPAR-α upregulation through the general control nonderepresible-2 (GCN2)/ activating transcription factor-6 (ATF6) pathway. Finally, in a genetic mouse model with BCAA catabolic defects, chronic accumulation of BCAA increased FAO in myocardial tissues and sensitized the heart to I/R injury, which could be reversed by adenovirus-mediated PPAR-α silencing.

**Conclusions**: We identify BCAA as an important nutrition regulator of myocardial fatty acid metabolism through transcriptional upregulation of PPAR-α. Chronic accumulation of BCAA, caused by either dietary or genetic factors, renders the heart vulnerable to I/R injury via exacerbating lipid peroxidation toxicity. These data support the notion that BCAA lowering methods might be potentially effective cardioprotective strategies, especially among patients with diseases characterized by elevated levels of BCAA, such as obesity and diabetes.

## Introduction

Ischemic heart disease (IHD) remains a leading cause of death in developed as well as developing countries 1. Although percutaneous coronary intervention can rapidly open the occluded coronary arteries, it paradoxically causes myocardial ischemia/reperfusion (I/R) injury, which is characterized by oxidative stress, cardiomyocyte death and contraction dysfunction 2-3. Unfortunately, effective therapeutic interventions for myocardial I/R injury are still lacking. The heart is a high-energy-consuming organ and energy substrate metabolism is essential to maintain the normal cardiac structure and function 4-6. Alterations in energy substrate utilization, in particular, glucose and fatty acid (FA) metabolism, have been recognized as critical determinants of myocardial vulnerability to I/R damage 7-8. Thus, modulation of cardiac substrate metabolism might be a promising therapeutic strategy for the management of I/R injury.

In addition to glucose and FAs, amino acids are important energy substrates to regulate cardiovascular pathophysiology. However, unlike glucose and FA metabolism, the regulatory role of amino acid metabolism in I/R process remains largely unknown. Branched chain amino acids (BCAA), consisting of valine, leucine and isoleucine, are a group of essential amino acids that can be oxidized in myocardial tissues 9. In mammalian animals, the metabolic homeostasis of BCAA is controlled by a series of BCAA catabolic enzymes. BCAA are firstly converted into corresponding branched chain α-keto acids (BCKA), which are subsequently decarboxylated by branched chain α-keto acid dehydrogenase (BCKDH) complex in the mitochondrion 9. As the rate-limiting enzyme of BCAA degradation, BCKDH complex is specifically dephosphorylated and activated by a mitochondrion-localized protein phosphatase-2C (PP2Cm) 10-11. Recently, numerous studies have revealed that elevation of BCAA/BCKA levels, due to dietary BCAA intake or PP2Cm genetic knockout (KO), directly contributes to the pathogenesis of a variety of cardiometabolic diseases, including heart failure 12, diabetes 13, and non-alcoholic fatty liver disease 14; however, the underlying mechanisms remain not fully understood.

It has recently been recognized that BCAA and their intermediate metabolites play important roles in the regulation of glucose and FA metabolism 14-15. For example, 3-hydroxyisobutyrate (a valine-derived metabolite) promotes trans-endothelial FA transport and causes lipid deposition in skeletal muscles 15. BCAA also stimulate triglyceride lipolysis in adipocytes and exacerbate high fat diet-induced hyperlipidemia 14. In the heart, chronic accumulation of BCAA disrupts glucose oxidation via suppressing mitochondrial pyruvate dehydrogenase (PDH) activity 8. These findings lead to a speculation that BCAA may affect cardiac vulnerability to I/R injury via modulating of myocardial substrate metabolism. Therefore, the present study aimed to: 1) systemically evaluate the impact of BCAA on cardiac energy substrate metabolism; 2) evaluate whether BCAA influence cardiac I/R vulnerability through modulation of cardiac energy substrate utilization; 3) if so, clarify the underlying molecular mechanisms involved.

## Materials and methods

### Animals and drugs

All animal studies were carried out in accordance with the National Institutes of Health Guidelines on the Use of Laboratory Animals and were approved by the Animal Care Committee of Air Force Medical University. PP2Cm global knockout (KO) mice are widely used as animal models with BCAA catabolic defects 10. KO mice were obtained and maintained as we previously described 13. Both KO and their wild-type (WT) littermates (aged from 10-12 weeks) were housed in a constant-temperature vivarium at 22°C with a 12-h light/dark cycle. Food and water were available ad libitum. BCAA mixture (weight ratio, leucine: valine: isoleucine=2:1:1; Sigma-Aldrich, St. Louis, MO, USA) were given into mice by oral gavage (1.5 mg/g/day) for 7 d before these mice received sham or I/R operation, as described by Li et al 8. Vehicle or Etomoxir (Eto) (5 mg/kg body weight, Sigma-Aldrich) was intraperitoneally injected at 15 min before I/R procedure 16. BCKA, including α-ketoisovaleric acid (αKIV), α-ketoisocaproate (αKIC) and α-keto-β-methylvalerate (αKMV), were commercially obtained from Sigma-Aldrich. A customized BCAA-free DMEM was used to exclude the impact of culture medium-contained BCAA. The detailed composition of BCAA-free DMEM is showed in Table S4.

### Myocardial I/R models

Myocardial I/R injury was induced as we previously performed 17. Briefly, mice were anesthetized with 2% isoflurane throughout the procedure. After a left thoracic incision and ribs exposure, the heart was mildly pumped out. A slipknot was tied on the left descending coronary artery to induce myocardial ischemia. After 30 min ischemia, the slipknot was loosened to reperfuse the myocardium for 3 h (to detect caspase-3 activation, superoxide production and LDH release) or 24 h (to detect cardiac function, infarct size and cardiac apoptosis). Mice in sham group underwent all the same procedure, except the knot tying.

### Intra-myocardial adenovirus injection and gene delivery

Adenovirus vectors carrying peroxisome proliferator-activated receptor alpha (Ppara)-specific short hairpin RNA (Ad-shPpara) or scramble control vectors (Ad-scramble) were constructed by Hanbio Co., Ltd (Shanghai, China). The sequences of shPpara and scramble were available in Table S1. Ad-shPpara and Ad-scramble vectors were delivered into the heart via intra-myocardial injection as we previously described 18. In brief, adenovirus vectors were diluted to 2.5×10^11^ particles/ml in phosphatase buffer (PBS). 25 μL adenovirus solutions were then injected into the left ventricular (LV) free wall using a Hamilton syringe (Hamilton Co. Reno, NV, USA). The intra-myocardial adenovirus injection was conducted as follows: 1) starting from apex and moving toward to the base in LV anterior wall; 2) at the upper part of LV anterior wall; 3) starting from apex and moving toward to base in LV posterior wall. After the injection, the heart was gently returned back to the thoracic cavity and carefully closed the incision. At 7 d after adenovirus injection, mice underwent sham or I/R operation as described above.

### Echocardiography

Mice were anesthetized by 2% isoflurane and ventricular function was determined by an echocardiographic imaging system (Vevo 2100, VisualSonics, Toronto, ON, Canada) at 24 h after sham or I/R operation. Two-dimensional echocardiographic views of the mid-ventricular short axis were collected at the section of the papillary muscle tips below the mitral valve. Subsequent calculation of the left ventricular ejection fraction (LVEF) and left ventricular fractional shortening (LVFS) were performed 19.

### Evaluation of cardiomyocyte apoptosis

To evaluate *in vivo* cardiomyocyte apoptosis, TdT-mediated dUTP nick-end labeling (TUNEL) staining (Roche, USA) was performed in heart tissue sections as the manual described. TUNEL/DAPI double-positive nuclei were counted as apoptotic cardiac myocytes. *In vitro* cardiomyocyte apoptosis was assessed by flow cytometry using the Annexin V-FITC/propidium iodide (PI) Apoptosis Detection kit (Beyotime, Beijing, China) according to the manufacturer's instructions.

### Evaluation of infarction size

At the end of a 24-h reperfusion, mice were anesthetized by 2% isoflurane and hearts were excised. The infarct size induced by I/R was measured by 5-triphenyltetrazolium chloride (TTC) and Evans blue double staining as we previously described 17.

### Adult mouse cardiac myocyte isolation

To isolate adult mouse cardiac myocytes, mice aged 10-12 weeks were anesthetized with 2% isoflurane and were fully heparinized with heparin. Hearts were carefully removed and placed into ice-cold PBS. Next, the heart was attached to the Langendorff system via the aorta and was fully perfused with perfusion solution (126 mmol/l NaCl, 4.4 mmol/l KCl, 18 mmol/l NaHCO_3_, 1 mmol/l MgCl_2_, 11 mmol/l glucose, 10 mmol/l 2,3-butanedione monoxime, 30 mmol/l taurine and 4 mmol/l HEPES) for 5 min. Thereafter, the heart was perfused by collagenase solution (perfusion solution with 0.1% bovine serum albumin, 0.025 mmol/l CaCl_2_ and 0.1% type II collagenase) for another 10 min. After digestion, the ventricle was triturated with 10 ml pipette at a slow speed and was filtered through a 100 μm filter. At last, cardiac myocytes were slowly adjusted to increasing concentrations of CaCl_2_ solution (range from 0.05 to 0.525 mmol/l) for 2 h before plating. Cardiac myocytes were seeded onto the laminin-coated plates overnight.

### Seahorse analysis

Metabolic flux experiments were performed in adult mouse cardiac myocytes because the metabolism of adult cardiac myocytes is largely different from neonatal cardiac myocytes. Briefly, adult mouse cardiac myocytes were seeded into laminin-coated XF24 Seahorse plates at a density of 10, 000 cells per well. Cardiac myocytes were cultured in a BCAA-free substrate-limited medium overnight before the analysis. The detailed composition of the BCAA-free substrate-limited medium is showed in Table S5. Oxygen consumption rate (OCR) and extracellular acidification rate (ECAR) were used to evaluate fatty acid oxidation (FAO), glucose oxidation and glycolysis in real-time when the appropriate substrates were added into or included in the assay medium. OCR and ECAR were measured using the Seahorse XF24 Extracellular Flux Analyzer (Seahorse Bioscience, North Billerica, MA, USA). Substrates or perturbation compounds were prepared in the assay medium as in the corresponding well and were added from the reagent ports automatically to the wells at the time as indicated.

### Preparation of palmitate-BSA conjugates

Palmitate was solubilized in 150 mmol/l sodium chloride by heating up to 65 °C in the water bath. BSA was dissolved in PBS and warmed up to 37 °C. Palmitate was then added into BSA at 37 °C with continuous stirring. When exogenous FAO flux was assessed by Seahorse, palmitate-BSA (0.175 mM) conjugate or BSA (0.03 mM) was added to appropriate wells.

### *In vitro* hypoxia/reoxygenation (H/R)

To simulate *in vivo* I/R injury, *in vitro* H/R models was established in neonatal rat ventricular myocytes (NRVMs). Briefly, NRVMs were isolated from Sprague-Dawley rats aged 1-3 d as we previously described 19. NRVMs were subjected to 9 h hypoxia and the following 3 h reoxygenation. To induce hypoxia, NRVMs were cultured in a hypoxia chamber with 95% N_2_ and 5% CO_2_ at 37 °C. H/R-induced lipid peroxidation, superoxide generation and cardiomyocyte apoptosis were systemically evaluated as the methods described.

### Determination of superoxide generation and lipid peroxidation

Superoxide generation was assessed by dihydroethidium (DHE, Sigma-Aldrich) staining in cultured NRVMs and freshly frozen heart tissue sections. Briefly, heart tissue sections or NRVMs were incubated with 40 μmol/l DHE for 1 h at room temperature, protected from light. The images were then obtained using a Leica laser scanning confocal microscope. Lipid peroxidation was determined by measuring malondialdehyde (MDA) and 4-hydroxynonenal (4-HNE) generation in NRVMs or heart tissues. MDA was measured using a commercial detection kit (S0131, Beyotime, Beijing, China) according to the instructions. 4-HNE content was measured using a commercially available kit (ab238538, Abcam, USA).

### Small interfering RNA (siRNA) transfection

NRVMs were routinely cultured. Specific siRNA of ATF6 (GenePharma, Shanghai, China) was obtained to knockdown ATF6 protein expression. Scrambled siRNA was also obtained from Genepharma. siRNA was transfected into cells by using Lipofectamine 2000 (Invitrogen, USA) according to manufacturers' instructions. Silencing efficacy was evaluated by immunoblotting. The sequences of siRNA were provided in Table S2.

### Real-time polymerase chain reaction (RT-PCR)

Total RNA was extracted from frozen heart tissues or cultured cells and RNA reverse-transcription were performed as we previously described 20. RT-PCR was conducted using a SYBR Green Master Mix (Cowin, Beijing, China). Primer sequences were listed in Table S3.

### Western blotting

Protein extraction and western blotting were performed as we previously described 21. In brief, proteins were electrophoresed on SDS-PAGE gels and transferred onto nitrocellulose membranes. Membranes were blocked in 5% non-fat milk and incubated with the following primary antibodies overnight at 4 °C. The primary antibodies were as follows: ACAA2 (1:1000, Abclonal, A15778), ACADM (1:1000, Abclonal, A1873), ATF6 (1:1000, Affinity, DF6009), β-actin (1:1000, Abclonal, AC004), Caspase-3 (1:1000, Cell Signaling Technology, 9662), CD36 (1:1000, Abclonal, A5792), Cleaved caspase-3 (1:1000, Cell Signaling Technology, 9661), CPT1B (1:1000, Abclonal, A6796), GCN2 (1:1000, Affinity, DF7801), p-GCN2 (1:1000, Affinity, AF8154) and PPAR-α (1:1000, Abclonal, A6697). After incubation in the secondary antibodies, blots were visualized using enhanced chemiluminescence (Millipore, USA) and scanned by ChemiDocXRS system (Bio-Rad Laboratory, Hercules, CA, USA).

### BCAA concentration measurement

Serum samples and cell supernatants were collected and immediately restored at -80 °C. BCAA concentration was determined with a commercially available BCAA detection kit (K564-100, Biovision, USA) as the protocol described. The absorbance value of each sample was measured at 450 nm in a microplate reader and calculated based on the standard curve.

### Statistical analysis

Data were analyzed with GraphPad Prism 8 statistic software and were presented as mean ± SEM. Statistical comparisons between groups were performed by Student's t-test for two groups and one-way ANOVA followed by Bonferroni Post-Hoc analysis for more than two groups. *P* < 0.05 was considered statistically significant.

## Results

### BCAA promote FAO in cardiac myocytes

**Figure 1:**
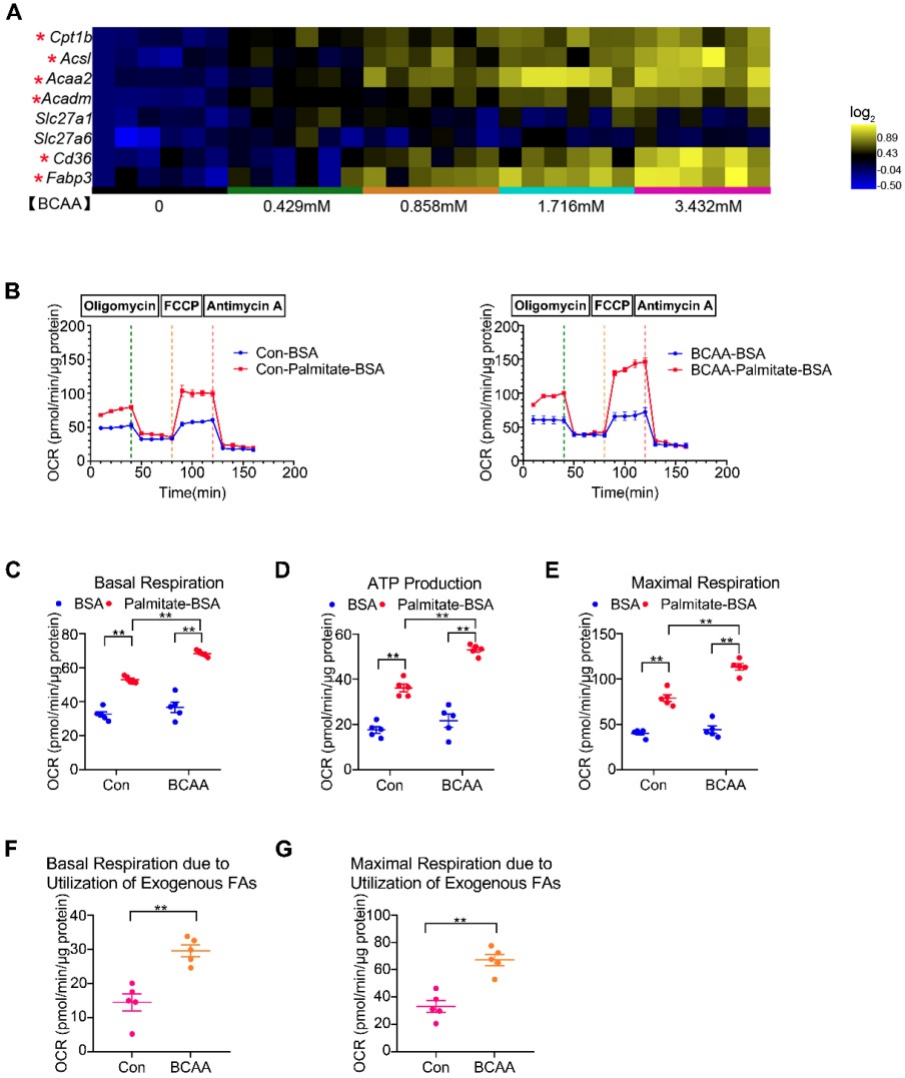
** BCAA promote FAO in cardiac myocytes**. (A) Adult mouse ventricular myocytes were isolated and treated with different concentrations of BCAA (0, 0.429 mM, 0.858 mM, 1.716 mM, 3.432 mM) for 12 h. Expression of Cpt1b, Acsl, Acaa2, Acadm, Slc27a1, Slc27a6, Cd36, Fabp3 mRNA by real-time PCR, normalized to β-actin (n=6). (B-G) Adult mouse cardiac myocytes were treated with or without BCAA (3.432 mM) for 12 h. FAO levels were determined by seahorse analyzer (n=4-5). (B) OCR curve of Con (No BCAA) group and BCAA group were determined. (C) Basal respiration (D) ATP production (E) maximal respiration (F) basal respiration due to exogenous palmitate-BSA and (G) maximal respiration due to exogenous palmitate-BSA were calculated according to instruction. (C-E) Data were analyzed by one-way ANOVA, followed by a Bonferroni post-hoc test. (F-G) Data were analyzed by Student's t test (two-tailed). * P<0.05, ** P<0.01. All values are presented as mean ± SEM.

Significant elevation of circulating BCAA levels has been observed in obese/diabetic mice, which are at high risk of cardiovascular diseases 13-14. Therefore, we attempted to determine how BCAA influence glucose and FA metabolism in cardiac myocytes. Adult mouse cardiac myocytes were incubated with increasing concentrations of BCAA (ranging from 0 to 3.432 mM). We found that high levels of BCAA inhibited mRNA expression of glucose oxidation enzymes and suppressed glucose-dependent oxygen consumption (Figure S1A, S2A and S3). Moreover, BCAA supplementation upregulated mRNA levels of glycolytic enzymes and augmented glycolysis in cardiac myocytes, which might be a compensatory phenomenon for BCAA-mediated glucose oxidation suppression (Figure S1B, S2B and S4). FAs are the predominant energy substrate in normal conditions. We next evaluated the impact of BCAA on FA metabolism in cardiac myocytes. Excessive BCAA remarkably increased mRNA levels of FA metabolism-related enzymes in a dose-dependent manner. These enzymes were involved in various important steps of FA metabolism, including FA transport (Cd36, Fabp3), esterification (Acsl), mitochondrial import (Cpt1b) and β-oxidation (Acaa2, Acadm) (Figure 1A and Figure S2C). Utilizing the Seahorse flux analyzer, we tested FAO capability of adult mouse ventricular myocytes with or without BCAA incubation (Figure 1B). Interestingly, when exogenous BSA or palmitate-BSA was added as energy substrate, BCAA-treated myocytes had a higher basal respiratory rate and ATP-linked respiration in comparison to the non-BCAA-treated group (Figure 1C-D). After the uncoupler FCCP injection, the BCAA treated group showed a significantly higher maximal respiration compared with the control (Figure 1E). Indeed, both basal and maximal mitochondrial respiration due to utilization of exogenous palmitate-BSA were elevated in BCAA-treated cardiac myocytes (Figure 1F-G). Together, our data indicate that high levels of BCAA enhance mitochondrial FA utilization in cardiac myocytes probably through transcriptionally upregulating FA metabolism-related enzymes.

### BCAA sensitize cardiomyocytes to H/R injury through increasing FAO levels

**Figure 2:**
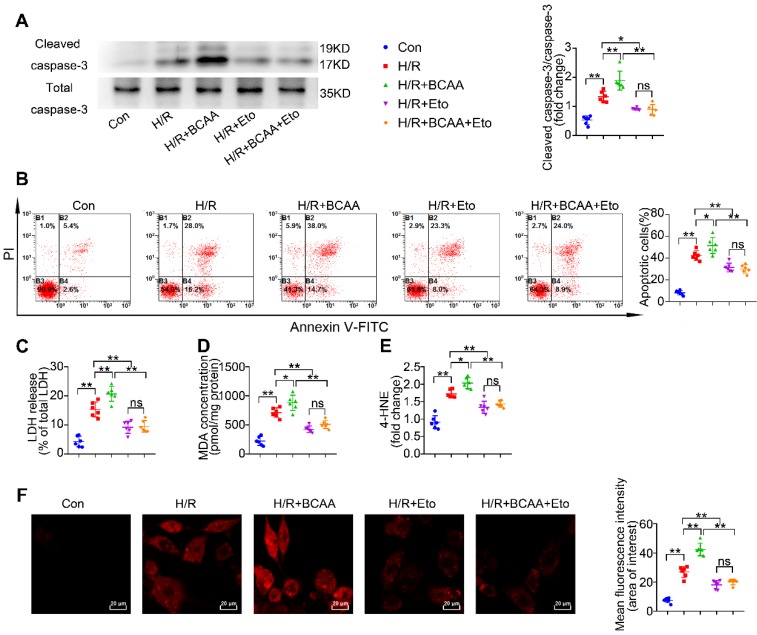
** BCAA exacerbate H/R injury via enhancing FAO**. NRVMs were isolated and subjected to 9 h/3 h H/R, with or without BCAA (3.432 mM) and Eto (10 nmol/L). (A) Cleavage of caspase-3 and total caspase-3 were determined by western blotting (n=6). (B) Apoptosis was analyzed by Annexin V-FITC flow cytometry (n=6). (C-E) LDH release, MDA and 4-HNE were determined as methods described (n=6). (F) Superoxide generation was assessed by DHE staining (n=6). Data were analyzed by one-way ANOVA, followed by a Bonferroni post-hoc test. * P<0.05, ** P<0.01.

Since BCAA suppress glucose oxidation but promote FAO in cardiac myocytes (Figure 1B-G), we next wondered whether BCAA worsen H/R injury via modulating cardiac energy substrate metabolism. In absence of H/R injury, increased concentrations of BCAA treatment did not induce obvious apoptosis, superoxide generation and lipid peroxidation in cultured NRVMs (Figure S5A-F). We found that BCAA treatment obviously increased the cleavage of caspase-3, a key mediator of cell apoptosis (Figure 2A). Flow cytometry analysis showed that BCAA exacerbated H/R-induced cardiomyocyte apoptosis (Figure 2B). LDH release assay also confirmed that BCAA aggravated H/R-induced cardiomyocyte damage (Figure 2C). Notably, these deleterious effects of BCAA on H/R injury were rescued by Eto, a specific inhibitor of FAO (Figure 2A to 2C). These data indicate that BCAA influence the vulnerability of cardiac myocytes to H/R injury due to FAO enhancement.

Inordinate FAO is known to increase lipid toxic metabolites and burst oxidative stress, thereby contributing to cardiac I/R injury. We tested the lipid toxic metabolites produced by FAO. In response to H/R, BCAA increased the accumulation of lipid peroxidation toxics, including MDA and 4-HNE (Figure 2D-E). BCAA supplementation also aggravated H/R-induced oxidative stress as indicated by increasing superoxide generation (Figure 2F). As expected, BCAA-encouraged lipid peroxidation and oxidative stress were rescued by Eto co-treatment. These data strongly suggest that BCAA render cardiac myocyte vulnerable to H/R injury via exacerbating FAO-related lipotoxicity.

### BCAA render the heart vulnerable to I/R injury via enhancing cardiac FAO levels

**Figure 3:**
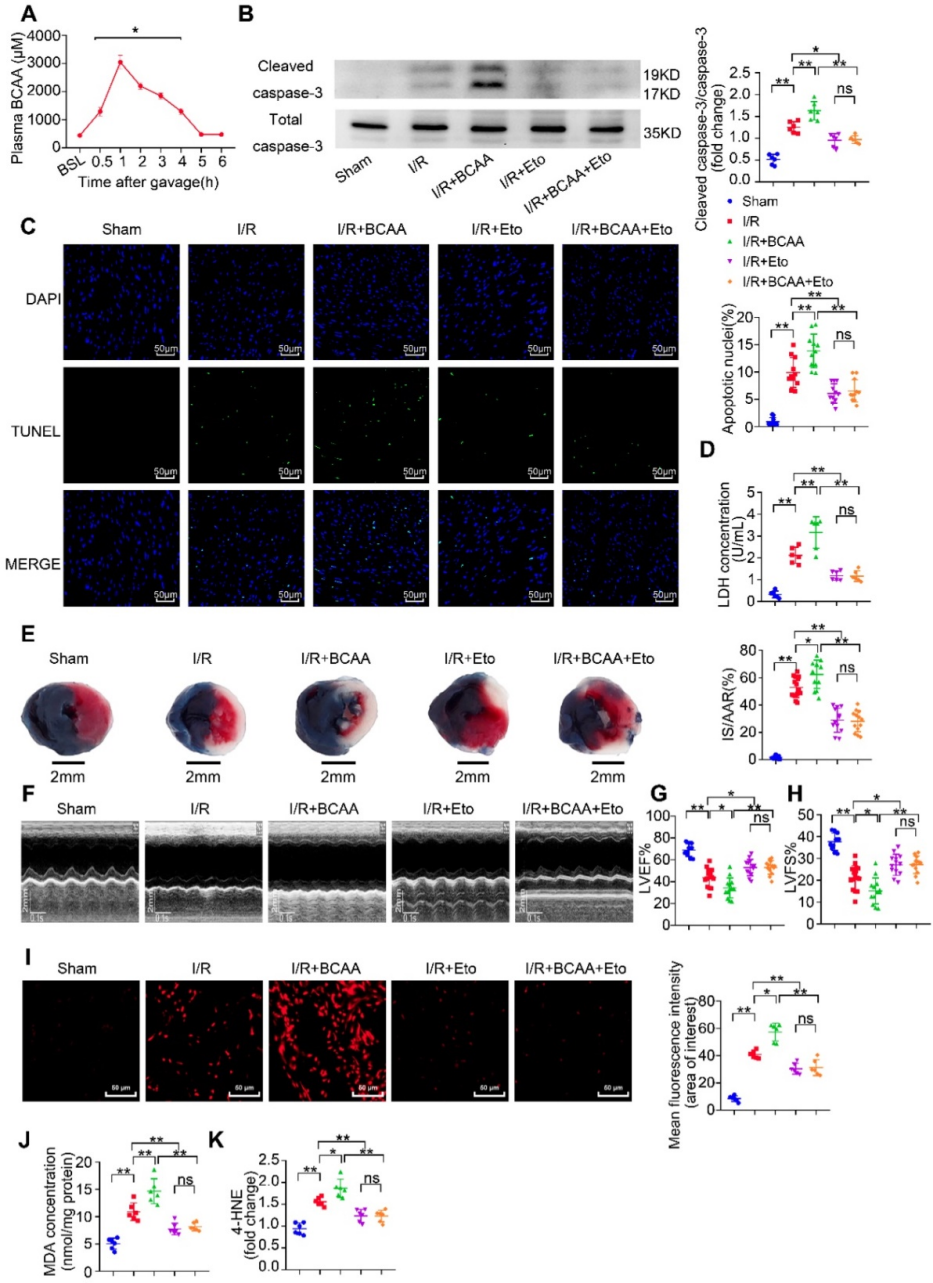
** BCAA worsen I/R injury, which can be rescued by inhibiting FAO**. (A) Serum BCAA concentrations at different time points after gavage (weight ratio, leucine: valine: isoleucine=2:1:1, 1.5 mg/g/day, n=6-8). (B-K) Vehicle or BCAA-supplemented (1.5 mg/g/day, 7 days) mice were treated with or without Eto (20 mg/kg body weight, i.p. injection 15 min before I/R surgery) under basal or I/R conditions. (B) Cardiac cleaved and non-cleaved caspase-3 by western blotting (n=6). (C) Representative cardiac apoptosis determined by TUNEL staining. Green fluorescence indicated TUNEL-positive cardiomyocyte nuclei; blue fluorescence showed total cardiomyocytes nuclei (n=10-15). Scale bar: 50 μm. (D) Cardiac apoptosis by LDH release assay (n=6). (E) Infarct area of heart tissue by Evans blue and tetrazolium chloride (TTC). The blue area represented unaffected heart tissue; white area showed infarcted tissue; red pus white area indicated tissue at risk (n=10-15). Scale bar: 2 mm. (F) Representative M-Mode echocardiographic images. (G and H) Echocardiographic assessment of LV ejection fraction and LV fractional shortening (n=10-15). (I) Superoxide production detected by DHE staining (n=6). Scale bar: 50 μm. (J and K) Lipid peroxidation determined by MDA and 4-HNE contents (n=6). (A) Data were analyzed by Student's t test. (B-K) Data were analyzed by one-way ANOVA, followed by a Bonferroni post-hoc test. * P<0.05, ** P<0.01. All values are presented as mean ± SEM.

In response to I/R, a metabolic conversion from FA to glucose utilization occurs and provides a compensatory cardioprotection 22. We next asked how elevated levels of BCAA affect cardiac I/R injury. To elevate circulating BCAA levels, mice were daily administered BCAA mixture (1.5 mg/g body weight) by gavage for 7 consecutive days. Prior to the I/R procedure, a final BCAA gavage was conducted and we found that serum BCAA levels were obviously elevated in the following 5 h (Figure 3A). Therefore, all I/R procedures were performed within 5 h upon the last BCAA gavage. In sham-operated animals, cardiac function was comparable between control and BCAA-gavage group assessed by echocardiography (Figure S5G-I).

Notably, compared with I/R group, BCAA-stimulated hearts showed remarkable upregulation of cleaved caspase-3 expression and LDH release (Figure 3B and 3D). Cardiomyocyte apoptosis determined by TUNEL/DAPI double staining was also increased in BCAA-treated mice (Figure 3C). 24 h after the I/R procedure, BCAA-treated mice showed an enlarged cardiac infarct size as determined by TTC/Evans blue staining and aggravation of ventricular dysfunction as determined by echocardiography (Figure 3E-H). BCAA treatment promoted generation of superoxide and lipid toxic metabolites such as MDA and 4-HNE (Figure 3I-K). Interestingly, BCAA-aggravated lipid peroxidation toxicity, oxidative stress, cardiomyocyte apoptosis and cardiac dysfunction were totally reversed by Eto-mediated FAO inhibition (Figure 3B-K). Collectively, these *in vivo* molecular, structural and functional experiments provide a clear-cut evidence that BCAA exacerbated myocardial vulnerability to I/R damage through promoting cardiac FAO.

### BCAA promote cardiomyocyte FAO via transcriptionally upregulating PPAR-α expression

**Figure 4:**
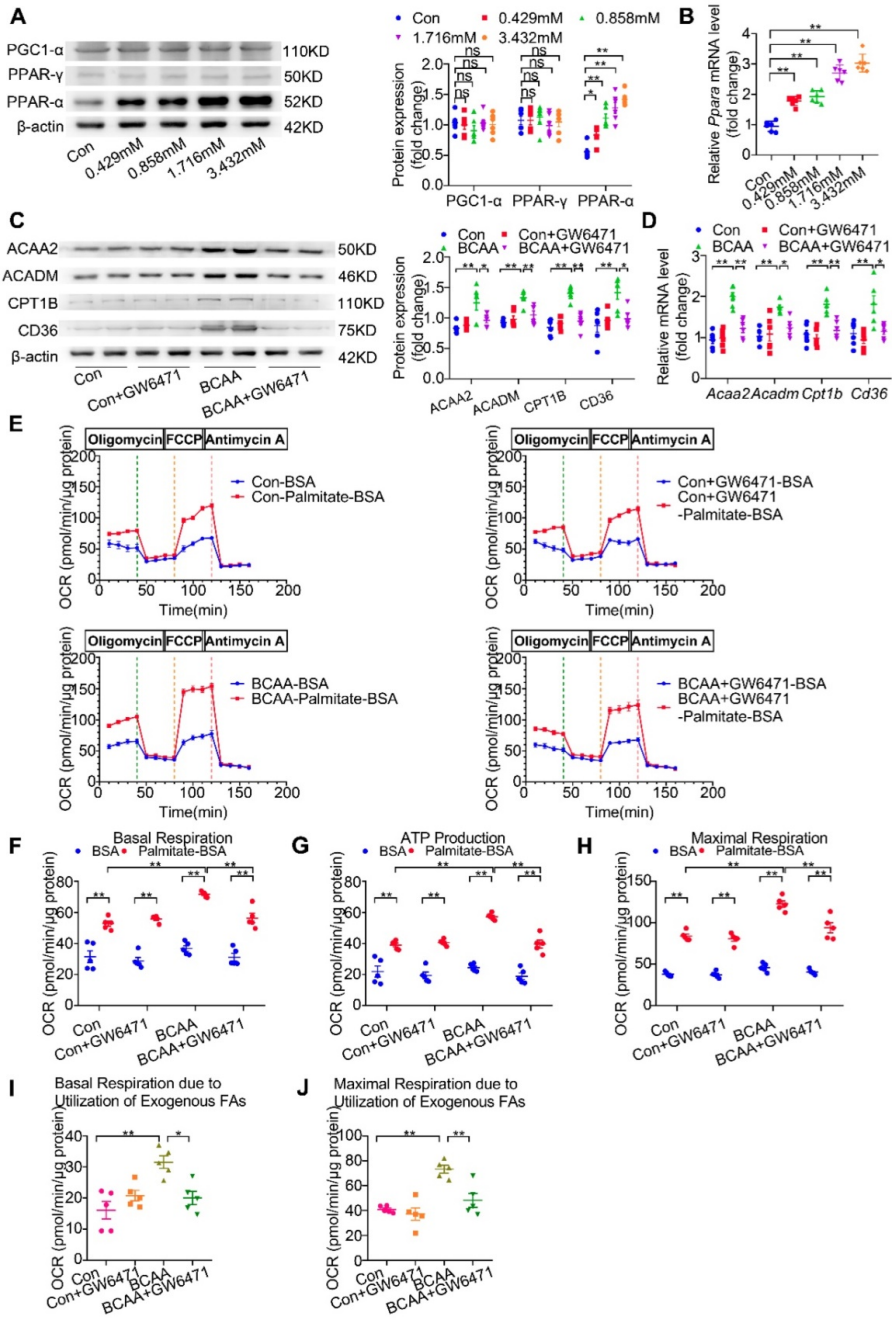
** BCAA upregulate PPAR-α and PPAR-α targeted genes**. (A to B) Adult mouse cardiac myocytes were treated with different concentrations of BCAA (0, 0.429 mM, 0.858 mM, 1.716 mM, 3.432 mM) for 12 h (n=6). (A) Expression of PGC1-α, PPAR-γ, PPAR-α in cardiomyocytes by western blotting. (B) Expression of Ppara in cardiomyocytes by real-time PCR. (C-J) Adult cardiac myocytes were treated with Vehicle (Con), GW6471, BCAA, BCAA+GW6471 for 12 h. (C and D) Expression of ACAA2, ACADM, CD36, CPT1B in cardiomyocytes by western blotting and real-time PCR (n=6). (E) OCR curve of adult cardiac myocytes treated with Vehicle (Con), GW6471, BCAA, BCAA+GW6471 were determined (n=5). (F) Basal respiration (G) ATP production (H) maximal respiration (I) basal respiration due to exogenous palmitate-BSA and (J) maximal respiration due to exogenous palmitate-BSA were calculated according to instruction. N=5 per group. * P<0.05, ** P<0.01. Data were analyzed by one-way ANOVA, followed by a Bonferroni post-hoc test. All values are presented as mean ± SEM.

The above-mentioned results show that BCAA upregulate FAO-related genes at the transcriptional levels and promote cardiac myocyte FAO levels. However, the underlying mechanisms involved remain to be elucidated. PPAR-α, peroxisome proliferator-activated receptor gamma (PPAR-γ) and peroxisome proliferator-activated receptor γ coactivator-1α (PGC1-α) have been identified as essential molecules to regulate FAO-related gene transcription 23-26. Thus, we wondered whether BCAA enhanced cardiac myocyte FAO levels through upregulating these proteins. After challenging cardiomyocytes with increasing doses of BCAA for 12 h, we evaluated PPAR-α, PPAR-γ and PGC1-α expression levels in cardiomyocytes. Our data showed a dose-dependent upregulation of PPAR-α in BCAA-treated cardiac myocytes, whereas the expression levels of PPAR-γ and PGC1-α were unchanged (Figure 4A). We also determined the acetylation and phosphorylation levels of PGC-1α when treated with different doses of BCAA in NRVMs to explore whether BCAA could lead to a post-translational modification on PGC-1α. Our results showed that the acetylation and phosphorylation level of PGC1-α remained unchanged after treatment with increasing doses of BCAA, indicating that PGC1-α might not be responsible for BCAA-enhanced FAO in cardiac myocytes (Figure S6). Notably, PPAR-α mRNA levels were concomitantly upregulated with its protein expression (Figure 4B), thereby suggesting that BCAA transcriptionally upregulated PPAR-α expression. Furthermore, both protein and mRNA expression levels of PPAR-α-targeted FAO enzymes were increased by BCAA supplementation (Figure 4C-D). Next, upregulation of FAO enzymes induced by BCAA was completely blocked by GW6471, a specific PPAR-α inhibitor (Figure 4C-D). To further confirm that BCAA-enhanced FAO was due to the upregulation of PPAR-α, we determined FAO levels using the Seahorse metabolic flux analyzer. As shown in Figure 4E-J, GW6471 alone did not affect cardiac myocyte FAO levels, when compared with control group. As expected, BCAA significantly enhanced mitochondrial FAO as indicated by higher palmitate-BSA-mediated mitochondrial oxygen consumption in cardiac myocytes. Intriguingly, the enhancement of FAO induced by BCAA was completely abolished by co-treatment with GW6471. Taken together, these data demonstrate that BCAA upregulate the expression of FAO-related enzymes and promote FAO by transcriptionally activating PPAR-α.

### Valine, leucine and their BCKA metabolites are responsible for BCAA-induced PPAR-α expression

**Figure 5:**
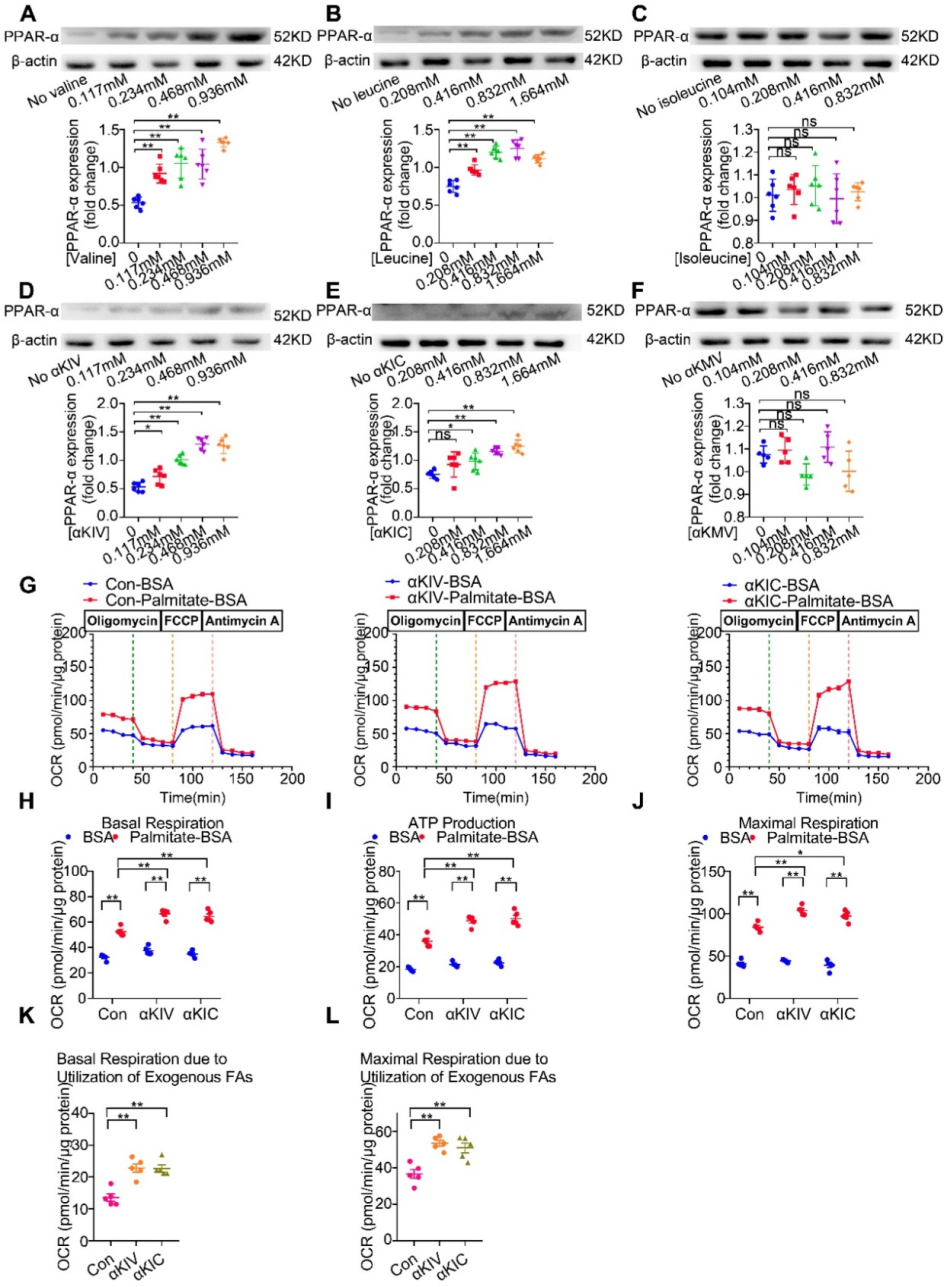
** The effects of BCAA/BCKA on FAO level and PPAR-α expression in cardiac myocytes**. (A) Expression of PPAR-α in the presence of increasing concentrations of valine (0, 0.117 mM, 0.234 mM, 0.468 mM, 0.936 mM) by western blotting (n=5-6). (B) Expression of PPAR-α at different concentrations of leucine (0, 0.208 mM, 0.416 mM, 0.832 mM, 1.664 mM) (n=5-6). (C) PPAR-α expression at different concentrations of isoleucine (0, 0.104 mM, 0.208 mM, 0.416 mM, 0.832 mM) (n=5-6). (D) Expression of PPAR-α in the presence of increasing concentrations of a-ketoisovaleric acid (αKIV) (0, 0.117 mM, 0.234 mM, 0.468 mM, 0.936 mM) (n=5-6). (E) Expression of PPAR-α at increasing concentrations of α-ketoisocaproic acid (αKIC) (0, 0.208 mM, 0.416 mM, 0.832 mM, 1.664 mM) (n=5-6). (F) PPAR-α expression at increasing concentrations of α-keto-β-methylvaleric acid (αKMV) (0, 0.104 mM, 0.208 mM, 0.416 mM, 0.832 mM) (n=5-6). (G to L) Adult mouse cardiac myocytes were treated with vehicle (Con), αKIV (0.936 mM) and αKIC (1.664 mM) for 12 h. FAO levels were determined by seahorse analyzer (n=5). (G) OCR curve of Con group, αKIV group and αKIC group were determined. (H) Basal respiration (I) ATP production (J) maximal respiration (K) basal respiration due to exogenous FAs and (L) maximal respiration due to exogenous FAs were calculated according to instruction. Data were analyzed by one-way ANOVA, followed by a Bonferroni post-hoc test. * P<0.05. ** P<0.01. All values are presented as mean ± SEM.

BCAA are a mixture of three essential amino acids, including valine, leucine and isoleucine. To determine which amino acid of BCAA is responsible for PPAR-α upregulation, cardiomyocytes were treated with different doses of valine, leucine and isoleucine, ranging from 1 to 8 folds of physiological levels, respectively. As shown in Figure 5A-C, valine and leucine dose-dependently increased PPAR-α protein expression in cultured cardiac myocytes. Valine and leucine induced PPAR-α upregulation even at their physiological blood levels. In contrast, isoleucine at doses up to 8-fold to the physiological concentrations failed to upregulate PPAR-α expression. Collectively, these data suggest that valine and leucine, but not isoleucine, are responsible for BCAA-induced PPAR-α expression. In mammalian cells, BCAA are first to undergo transamination to generate corresponding BCKA derivatives via branched-chain amino acid aminotransferase (BCAT) 27. Compared with other organs, the activity of BCAT is the highest in the heart 28. More importantly, in cardiovascular and metabolic diseases with BCAA catabolic defects, BCKA also elevate in the serum and directly contribute to disease progression 29. Thus, additional studies were conducted to identify the effect of BCKA on PPAR-α expression and FAO in cardiomyocytes. Similar to valine and leucine, valine-deprived αKIV and leucine-deprived αKIC dose-dependently upregulated PPAR-α expression in cardiomyocytes (Figure 5D-E). However, isoleucine-deprived αKMV had no obvious effect on PPAR-α expression (Figure 5F). Combined, these data suggest that valine, leucine and their BCKA products account for BCAA-induced PPAR-α upregulation. Consistently, using seahorse metabolic flux analyzer, we found αKIV and αKIC enhanced FAO in cardiac myocytes as indicated by increased palmitate-BSA-induced oxygen utilization (Figure 5G-L).

In contrast, αKMV had no effect on the palmitate-BSA-induced oxygen consumption, suggesting that αKMV did not promote FAO in cardiac myocytes (data not shown). Thus, we showed for the first time that valine, leucine and their corresponding BCKA derivatives are important nutritional regulators of PPAR-α and FAO in cardiac myocytes.

### BCAA increase PPAR-α expression through amino acid sensing GCN2/ATF6 pathway

**Figure 6:**
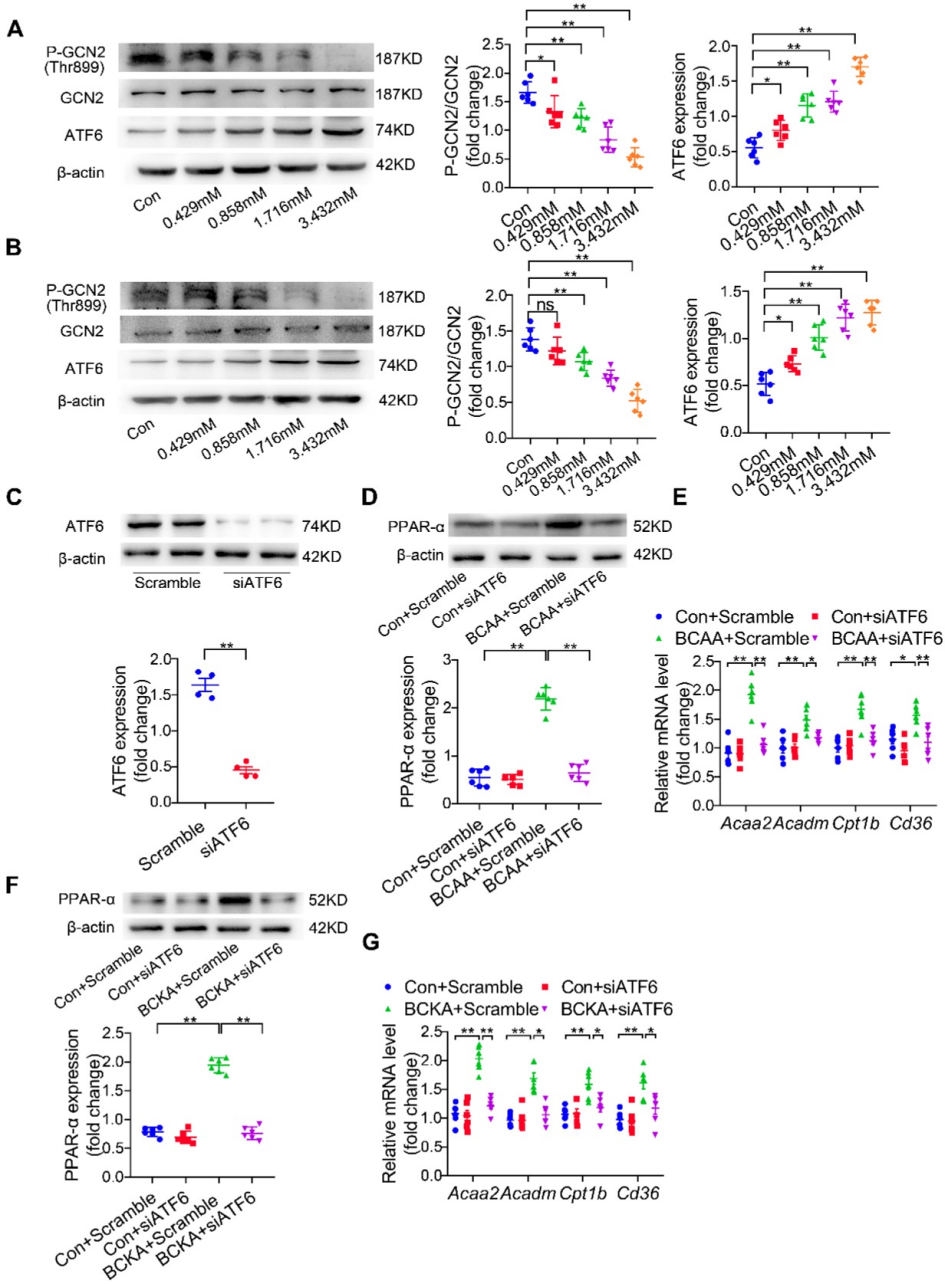
** BCAA increase PPAR-α expression in a GCN2/ATF6 pathway-dependent manner.** (A) Expression of p-GCN2, GCN2 and ATF6 in the presence of increasing concentrations of BCAA (0, 0.429 mM, 0.858 mM, 1.716 mM, 3.432 mM) by western blotting (n=6). (B) Expression of p-GCN2, GCN2 and ATF6 in the presence of increasing concentrations of BCKA (0, 0.429 mM, 0.858 mM, 1.716 mM, 3.432 mM) by western blotting (n=6). BCKA mixture is composed of αKIC, αKIV and αKMV (weight ratio, αKIC: αKIV: αKMV= 2:1:1). (C) NRVMs were treated with control siRNA and ATF6 siRNA. 48 h after transfection, expression of ATF6 was determined by western blotting (n=4). (D-E) ATF6 siRNA transferred NRVMs were treated with or without BCAA (3.432 mM) (n=6). (D) PPAR-α expression was determined by western blotting. (E) Expression of Acaa2, Acadm, Cd36 and Cpt1b by real-time PCR. (F-G) ATF6 siRNA transferred NRVMs were treated with or without BCKA (3.432 mM) (n=6). (F) PPAR-α expression was determined by western blotting. (G) Expression of Acaa2, Acadm, Cd36 and Cpt1b by real-time PCR. (C) Data were analyzed by Student's t test. (A-B) and (D-G) Data were analyzed by one-way ANOVA, followed by a Bonferroni post-hoc test. * P<0.05, ** P<0.01. All values are presented as mean ± SEM.

Another challenging question involves how BCAA/BCKA induces PPAR-α expression in cardiomyocytes. In addition to being materials to synthetize protein or other non-essential amino acids, BCAA are important intracellular signaling transduction molecules via regulating amino acid sensing pathways 27. Elevated levels of amino acids lead to inactivation of general control nonderepresible-2 (GCN2), a protein involved in sensing intracellular amino acid deprivation 30. Moreover, loss of GCN2 leads to the upregulation of activating transcription factor-6 (ATF6), an indispensable molecule for PPAR-α transcription 31-32. Thus, we asked whether BCAA could upregulate PPAR-α expression in a GCN2/ATF6-dependent manner. Our data showed that BCAA treatment inactivated GCN2 as indicated by its decreased phosphorylation at the Thr899 site (Figure 6A). Furthermore, BCAA dose-dependently upregulated ATF6 expression (Figure 6A). Similar to BCAA, the mixture of BCKA (αKIV 0.936 mmol/l, αKIC 1.664 mmol/l and αKMV 0.832 mmol/l) also inactivated GCN2 and upregulated ATF6 expression in a dose-dependent manner (Figure 6B). Additional studies were conducted to test whether ATF6 mediated BCAA-induced PPAR-α upregulation. Cardiomyocytes were transfected with ATF6-specific siRNA, which resulted in obvious downregulation of ATF6 expression (Figure 6C). ATF6 knockdown significantly blocked BCAA-induced upregulation of PPAR-α and its downstream FAO-related genes (Figure 6D-E). Consistently, ATF6 silencing suppressed BCKA-induced upregulation of PPAR-α and its target genes (Figure 6F-G). Collectively, we conclude that BCAA/BCKA upregulate PPAR-α expression through regulating GCN2/ATF6 signaling pathway.

### Defective BCAA catabolism sensitizes cardiomyocytes to H/R injury, which is rescued by PPAR-α silencing

**Figure 7:**
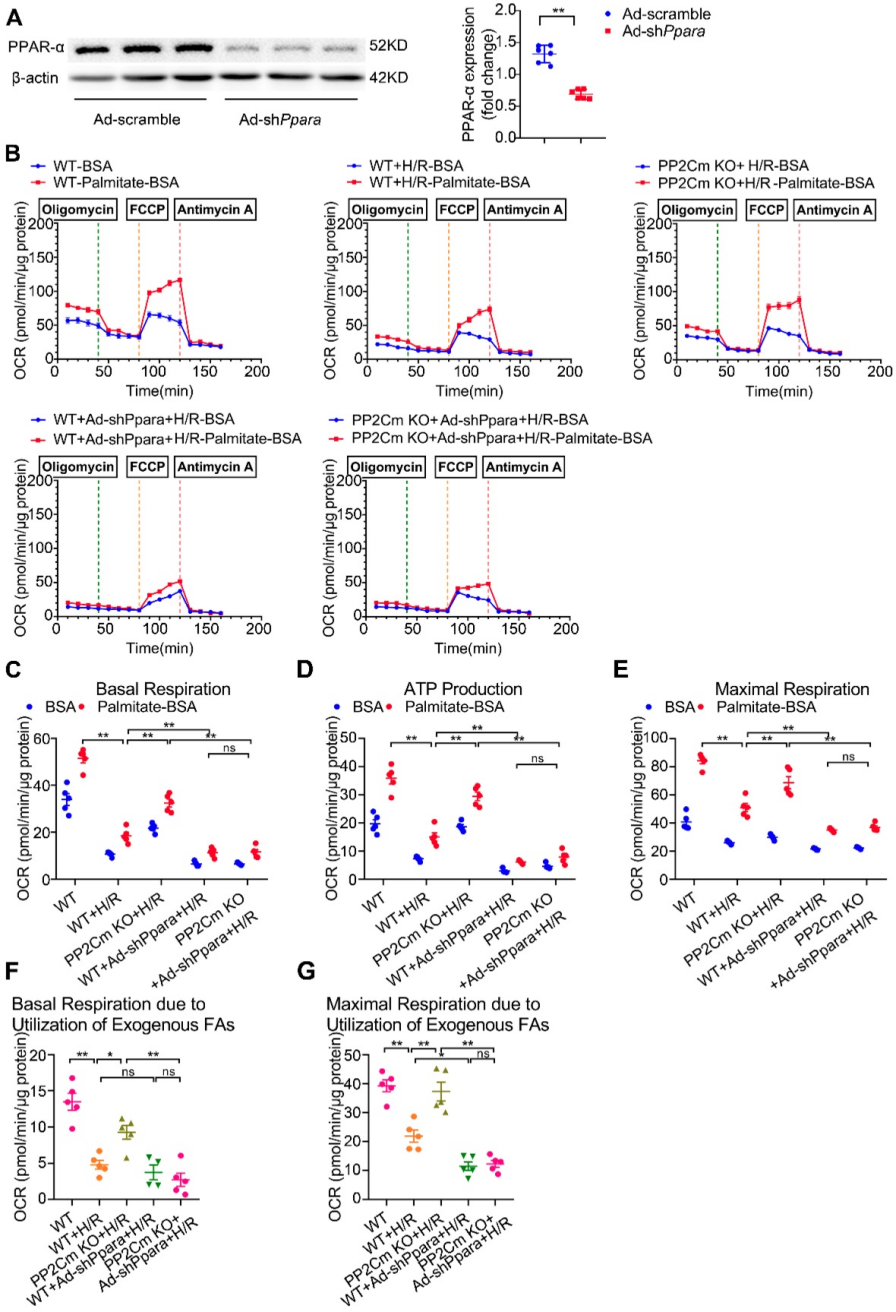

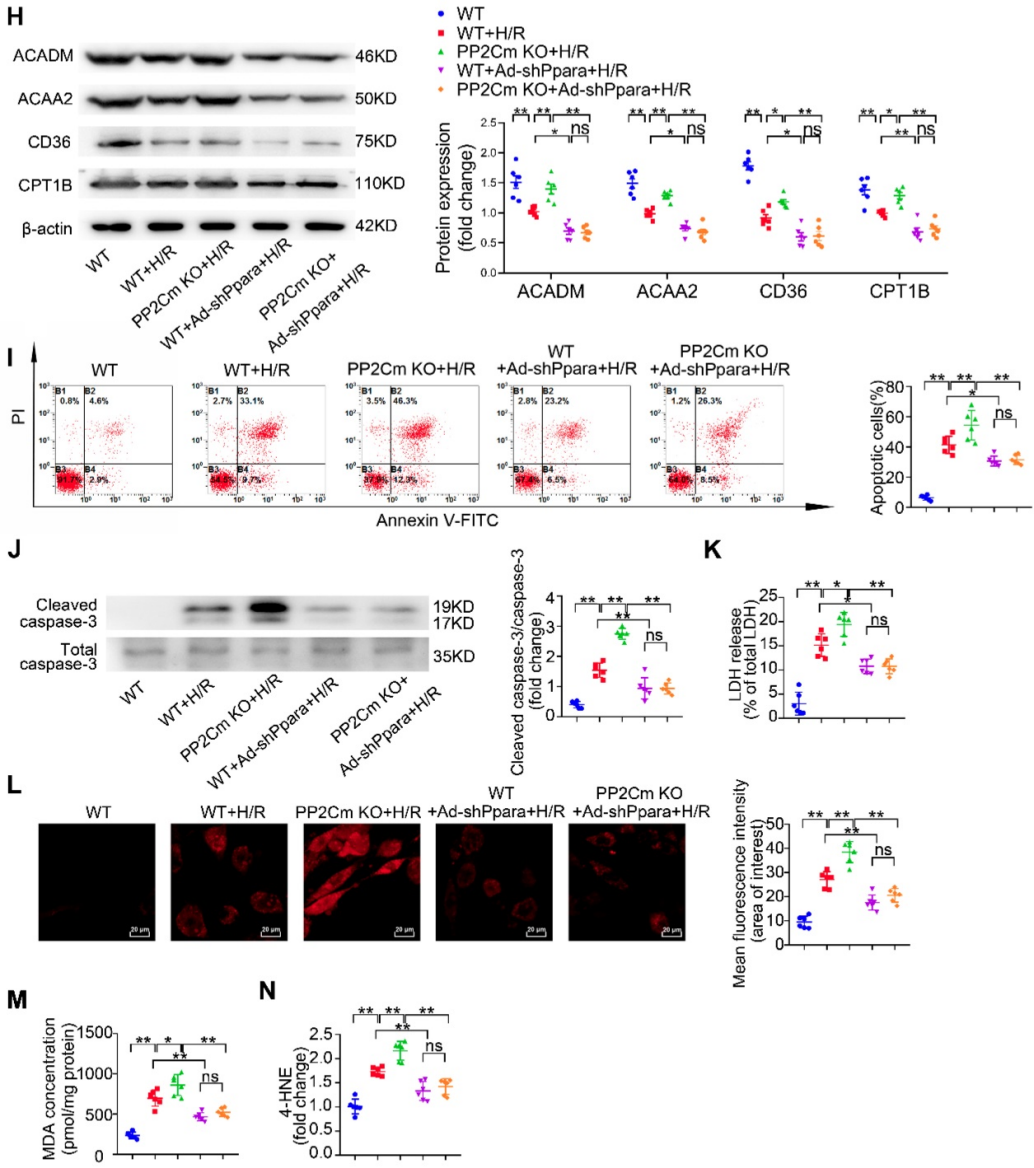
** PPAR-α knockdown suppresses myocardial FAO levels and protects PP2Cm KO cardiomyocytes against injury following H/R**. (A) Expression of PPAR-α by western blotting in cardiomyocytes with or without shPpara adenovirus infection (n=6). (B to N) Ventricular myocytes isolated from WT mice or PP2Cm KO mice were infected with scrambled or shPpara adenovirus for 48 h with or without H/R injury. (B to G) FAO levels were determined by seahorse analyzer (n=4-5). (B) OCR curve treated as mentioned above were determined. (C) Basal respiration (D) ATP production (E) maximal respiration (F) basal respiration due to exogenous FAs and (G) maximal respiration due to exogenous FAs were calculated according to instruction. (H) Expression of ACAA2, ACADM, CD36, CPT1B in cardiomyocytes by western blotting (n=6). (I) Annexin V and propidium iodide (PI) staining by flow cytometry for cardiomyocyte apoptosis determination (n=6). (J) Cleaved and non-cleaved caspase-3 by western blotting (n=6). (K) Cell death assessed by LDH release (n=6). (L) Superoxide production detected by DHE staining (n=6). Scale bar: 20 μm. (M and N) Lipid peroxidation determined by MDA and 4-HNE contents (n=6). Data were analyzed by one-way ANOVA, followed by a Bonferroni post-hoc test. * P<0.05, ** P<0.01. All values are presented as mean ± SEM.

We next investigated whether PPAR-α-mediated metabolic reprogramming would affect the I/R vulnerability of hearts with BCAA catabolic defects. In PP2Cm knockout (KO) mice, BCKDH (the rate-limiting enzyme complex for BCAA degradation) is inactivated due to its sustained phosphorylation. Thus, PP2Cm KO mice have been widely used as a mouse model with defective BCAA catabolism 8, 12. In our study, cardiac myocytes were isolated from WT or PP2Cm KO mice. We found that, in response to H/R injury, PP2Cm deficient cells showed higher palmitate-induced mitochondrial oxygen consumption than WT group (Figure 7B-G), suggesting that defective BCAA catabolism augments FAO in cardiac myocytes. PPAR-α was silenced by adenovirus-carrying short hairpin RNA (Ad-shPpara, Figure 7A). Notably, PP2Cm KO-enhanced FAO was significantly diminished by Ad-shPpara transfection (Figure 7B-G). In PP2Cm KO cardiomyocytes, PPAR-α targeted FAO genes, including Acadm, Acaa2, Cd36 and Cpt1b, were upregulated compared with WT group (Figure 7H). PPAR-α shRNA abolished the expression of PP2Cm KO-induced upregulation of FAO-related genes. These data further support that cardiac myocytes with defective BCAA catabolism have higher FAO levels due to PPAR-α upregulation. In absence of H/R injury, PP2Cm KO failed to induce apoptosis, lipid peroxidation or superoxide generation (Figure S7A-F). However, additional studies showed that PP2Cm deficient cardiomyocytes were more susceptible to H/R-induced apoptosis (Figure 7I-K). PP2Cm KO mice exacerbated H/R-induced oxidative stress (Figure 7L) and lipid peroxidation toxicity (Figure 7M-N). These deleterious effects of PP2Cm KO on H/R injury were obviously rescued by PPAR-α silencing (Figure 7I-N). Collectively, these data demonstrate that, similar to exogenous BCAA supplementation, chronic BCAA accumulation due to endogenous BCAA catabolic defects sensitize the cardiomyocytes to H/R injury through enhancing PPAR-α-mediated FAO and lipid peroxidation toxicity.

### Exacerbated I/R injury in the PP2Cm KO heart is rescued by PPAR-α knockdown

**Figure 8:**
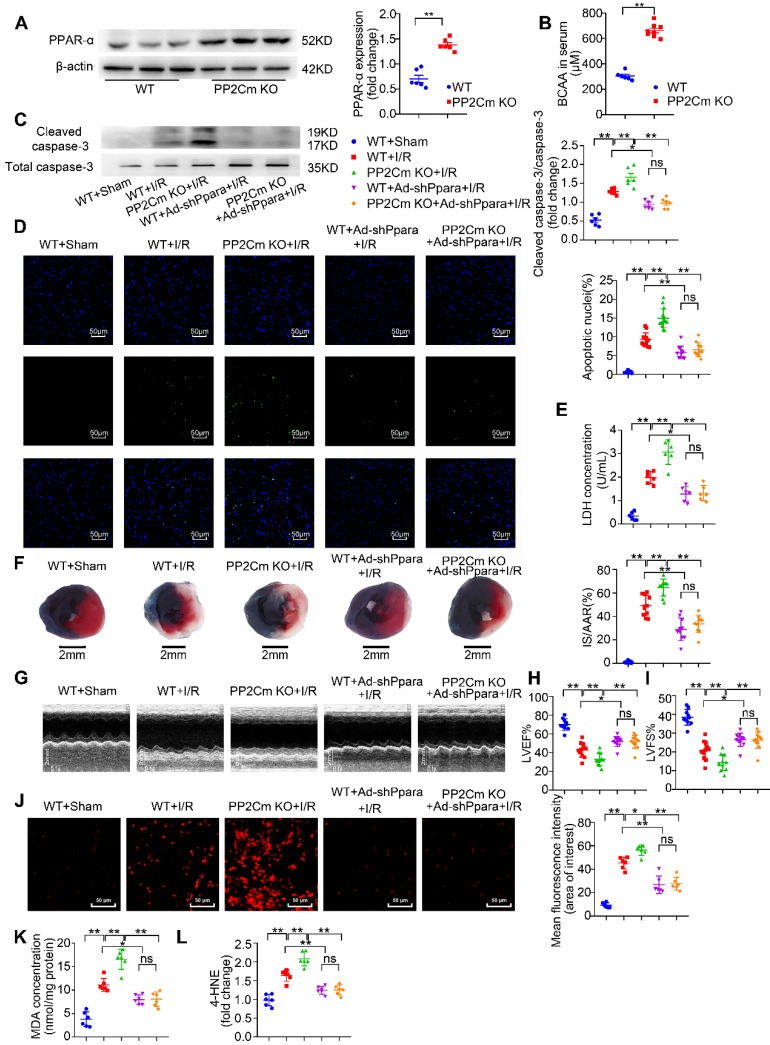
** Exacerbated I/R injury in the PP2Cm KO heart is rescued by PPAR-α knockdown**. (A) Expression of PPAR-α were determined by western blotting in WT mice and PP2Cm KO mice (n=6). (B) WT and PP2Cm KO serum BCAA were determined (n=6-8). (C to L) WT mice or PP2Cm KO mice were received intra-myocardial injection with scrambled or shPpara adenovirus 7 days before sham or I/R operation. (C) Cleaved and non-cleaved caspase-3 by western blotting (n=6). (D) Cardiac apoptosis determined by TUNEL staining (n=10-15). Scale bar: 50 μm. (E) Cardiac death by LDH release assay (n=6). (F) Infarct area of heart tissue by Evans blue and TTC (n=10-15). Scale bar: 2 mm. (G) Representative M-Mode echocardiographic images. (H and I) Echocardiographic assessment of LV ejection fraction and LV fractional shortening (n=10-15). (J) Superoxide production detected by DHE staining (n=6). Scale bar: 50 μm. (K and L) Lipid peroxidation determined by MDA and 4-HNE (n=6). (A-B) Data were analyzed by Student's t test. (C-L) Data were analyzed by one-way ANOVA, followed by a Bonferroni post-hoc test. * P<0.05, ** P<0.01. All values are presented as mean ± SEM.

Chronic BCAA accumulation due to their catabolic defects often occurs in cardiometabolic diseases, such as heart failure, obesity and diabetes 12-14. In these diseases, the heart is more vulnerable to I/R injury 33. Therefore, utilizing PP2Cm KO mice, we further determined whether defective BCAA catabolism affected I/R vulnerability and investigated the involvement of PPAR-α. In basal conditions, cardiac function in PP2Cm KO group was comparable to WT group (Figure S7G-I). PPAR-α expression was increased in PP2Cm KO hearts (Figure 8A). Similar to exogenous BCAA accumulation by oral gavage, PP2Cm KO resulted in endogenous BCAA accumulation as indicated by higher serum BCAA levels (Figure 8B). Chronic accumulation of BCAA exacerbated I/R-induced caspase-3 activation, cardiomyocyte apoptosis and LDH release (Figure 8C-E). Consistently, PP2Cm KO hearts had larger infarct sizes and worse ventricular function in response to I/R injury (Figure 8F-I). These data establish a causative role of defective BCAA catabolism in the regulation of myocardial I/R vulnerability.

Next, we asked whether defective BCAA catabolism could render the heart vulnerable to I/R injury due to upregulation of PPAR-α. WT and PP2Cm KO hearts were transfected with adenovirus vectors carrying PPAR-α shRNA (Ad-shPpara). We found that intra-cardiac Ad-shPpara injection obviously reduced PPAR-α expression in cardiac tissue (Figure S8). Moreover, compared with the KO heart, PPAR-α silencing rescued BCAA catabolic defects-aggravated cardiomyocyte apoptosis, infarction and ventricular dysfunction (Figure 8C-I). In PP2Cm KO hearts, chronic accumulation of BCAA exacerbated superoxide generation and lipid peroxidation toxicity (Figure 8J-L), which was reversed by adenovirus-mediated PPAR-α knockdown. Taken together, these data demonstrate that defective BCAA catabolism sensitize the heart to I/R injury via upregulating PPAR-α expression.

## Discussion

In the present study, we have several important observations. First, we confirm that BCAA are important endogenous nutrition regulator of cardiac FAO. It has been reported that BCAA suppress cardiac glucose oxidation via inhibiting PDH activity 8. However, FAs are the dominant energy substrate and provide 50-70% of total ATP for the heart in normal conditions 34-35. Therefore, it is of great significance to clarify the impact of BCAA on cardiac FA metabolism. Utilizing the Seahorse metabolic flux analyzer, we for the first time reveal that BCAA enhance endogenous and exogenous FA utilization in adult cardiac myocytes. We also confirm that valine and leucine, but not isoleucine, are responsible for BCAA-induced FAO enhancement. Most amino acids are mainly degraded in the liver whereas BCAA are first degraded in extra-hepatic organs, such as the heart and the skeletal muscle 28, 36. We next evaluate the impact of BCAA downstream metabolites BCKA on cardiac FAO. The present study reveals that valine-derived α-KIV and leucine-derived αKIC, but not isoleucine-derived α-KMV, enhance FAO in cardiac myocytes. Collectively, these data reveal that BCAA and their metabolites BCKA act as an important endogenous nutrition regulator of FAO in cardiac myocytes.

Secondly, we have identified PPAR-α as an indispensable transcriptional factor for BCAA-enhanced FAO. PPAR-α is a critical regulator of FA metabolism via transcriptionally regulating the expression levels of FAO-related enzymes 24. In the present study, we confirm that BCAA/BCKA transcriptionally upregulate PPAR-α expression. Pharmacological inhibition of PPAR-α by GW-6471 blocks BCAA/BCKA-induced FAO enhancement, revealing an indispensable role of PPAR-α in this process. Furthermore, we also clarify that BCAA/BCKA upregulate PPAR-α expression through the amino acid sensing GCN2/ATF6 pathway. Amino acid starvation leads to phosphorylation and activation of GCN2 30. Therefore, elevated levels of intracellular amino acids cause dephosphorylation and inactivation of GCN2. ATF6 is a transcription factor activated by endoplasmic reticulum stress and is essential for PPAR-α transcription 32, 37. In the present study, we find that BCAA/BCKA inactivate GCN2 and upregulate ATF6, thereby promoting PPAR-α transcription. This novel signaling pathway links intracellular amino acid sensing and FA metabolism in cardiac myocytes. Collectively, these data for the first time demonstrate that BCAA/BCKA promote FAO through the GCN2/ATF6/ PPAR-α pathway.

Thirdly, we have provided a clear-cut evidence that chronic accumulation of BCAA/BCKA, no matter induced by dietary or genetic factors, increase the vulnerability to I/R injury due to the enhancement of FAO. Compared with glucose oxidation, FAO results in higher oxygen consumption, lower ATP production efficacy and much more lipotoxic product accumulation. Thus, in response to I/R injury, the metabolic shift from FA to glucose utilization is considered as a compensatory protection 5. In the heart, downregulation of PPAR-α has been shown to exert cardioprotection against I/R injury through preventing lipid peroxidation toxicity 38-39. We have recently observed that PP2Cm deficient heart is more susceptible to I/R injury due to increased mitochondrial superoxide generation; however, the underlying mechanisms are totally unknown 40. Here we further demonstrate that chronic accumulation of BCAA renders the heart vulnerable to I/R injury, which could be rescued by FAO inhibition or PPAR-α silencing. These results reveal that elevated levels of BCAA due to excessive BCAA intake or defective BCAA catabolism regulate metabolic substrate preference and determine cardiac vulnerability to I/R. Defective BCAA catabolism and elevated BCAA/BCKA levels are often seen in the development of cardiometabolic diseases, such as heart failure, diabetes, obesity and non-alcoholic fatty liver disease 12-14. Patients with these above diseases are more susceptible to myocardial I/R injury. Therefore, the present study calls for the caution that elevated levels of BCAA should be intervened to reduce myocardial vulnerability to I/R injury in these patients.

In summary, the present studies for the first time reveal that BCAA/BCKA is important nutrition regulators of cardiac energy substrate preference. BCAA/BCKA enhances cardiac FAO levels via transcriptionally upregulating PPAR-α expression, thereby exacerbating lipid peroxidation toxicity and cardiac vulnerability to I/R injury. Given the fact that BCAA catabolism is impaired in multiple cardiometabolic diseases, this study suggests that targeting BCAA catabolism might be a brand-new strategy to manage cardiac vulnerability to I/R injury among patients with diseases characterized by defective BCAA catabolism.

## Supplementary Material

Supplementary figures and tables.

## Abbreviations

Acaa: acetyl-CoA acetyltransferase
Acadm: medium-chain specific acyl-CoA dehydrogenase, mitochondrial
Acsl: long-chain acyl-CoA synthetase
ATF6: activating transcription factor 6
BCAA: branched chain amino acids
BCAT: branched-chain amino acid aminotransferase
BCKA: branched chain α-keto acid
BCKDH: branched chain α-keto acid dehydrogenase
Cd36: fatty acid translocase
Cpt1b: carnitine palmitoyltransferase 1B
DHE: dihydroethidium
ECAR: extracellular acidification rate
Eto: etomoxir
FA: fatty acid
FAO: fatty acid oxidation
GCN2: general control nonderepresible-2
H/R: hypoxia/reoxygenation
HNE: hydroxynonenal
IHD: ischemia heart disease
I/R: ischemia/reperfusion
KO: knockout
LVEF: left ventricular ejection fraction
LVFS: left ventricular fractional shortening
MDA: malondialdehyde
NRVMs: neonatal rat ventricular myocytes
OCR: oxygen consumption rate
PDH: pyruvate dehydrogenase
PGC1-α: peroxisome proliferator-activated receptor γ coactivator-1α
PP2Cm: mitochondrion-localized protein phosphatase-2C
PPAR-α: peroxisome proliferation-activated receptor alpha
PPAR-γ: peroxisome proliferator-activated receptor gamma
TTC: 5-triphenyltetrazolium chloride
TUNEL: TdT-mediated dUTP nick-end labeling
WT: wild type
