# Supplementary material for: Branched chain amino acids exacerbate myocardial ischemia/reperfusion vulnerability via enhancing GCN2/ATF6/PPAR-α pathway-dependent fatty acid oxidation
Source: Theranostics. 2020 Apr 27;10(12):5623–40. doi: 10.7150/thno.44836 (PMC7196282; doi:10.7150/thno.44836)
Supplement: Supplementary file 1 — Supplementary figures and tables. [file thnov10p5623s1.pdf]

Table S1. Sequences of shRNA.

shRNA		Sequence
Ppara	Top strand	tcgagGTCACACAATGCAATTCGCTTTGGAATTCAAGA<br>GATTCCAAAGCGAATTGCATTGTGTGATTTTTT
	Bottom strand	agcttAAAAAATCACACAATGCAATTCGCTTTGGAATC<br>TCTTGAATTCCAAAGCGAATTGCATTGTGTGACc
Scramble	Top strand	GATCCGTTCTCCGAACGTGTCACGTAATTCAAGAGA<br>TTACGTGACACGTTCGGAGAATTTTTTC
	Bottom strand	AATTGAAAAAATTCTCCGAACGTGTCACGTAATCTC<br>TTGAATTACGTGACACGTTCGGAGAACG

Table S2. Sequences of siRNA

siRNA	Sequence	
ATF6 siRNA	Sense	GGGCAGGAUUAUGAAGUAATT
	Antisense	UUACUUCAUAAUCCUGCCCTT
Scramble siRNA	Sense	UUCUCCGAACGUGUCACGUTT
	Antisense	ACGUGACACGUUCGGAGAATT

Table S3. Sequences of Primers used in RT-PCR

Gene symbol	Forward 5' to 3'	Reverse 5' to 3'
Cpt1b	TACACGCATCCCAGGCAAAG	CGAGCCCTCATAGAGCCAAAC
Acsl1	TTCGCAGTGGCATCGTCAG	TGTGATCATCAGCCGGACTTTC
Acaa2	ACACCTGGTTCACGAGTTAAG	GTTCTGGATGATCAGGGAGATG
Acadm	GCTACAAGGTCCTGAGAAGTG	CTCCGTCAACTCGAAGCTAAA
Slc27a1	CAGACGGACGTGGCTGTGTA	GCTGGGCATAGGATGCAAGAA
Slc27a6	CATCATGTCACCTCAAGCCTCAA	TCATCAGCTGTGCAGCCAAAC
Cd36	TCAGAACCTATCGAAGGCTTGAATC	AGCTGGCTTGACCAGTATGTTGAC
Fabp3	TAGCATGACCAAGCCGACCA	ACCAGTTTGCCTCCGTCCAG
Pdha1	TCAATGCACATGTACGCCAAGA	TTATACTTGCAGGCCAGAGCAATTC
Pdhb	CACATCACTGTAGTTGCCCATTC	ATAGCTTCAATGTCCATTGGTCTG
Idh1	GAGGCTTCATCTGGGCCTGTAA	CATGGGCAGCCTCTGCTTCTA
Ogdh	CTGGCCAGGGTATCGTGTATGAG	CATCCGAGGGTCTGTGGTGA
Cs	TGGCCCAACGTAGATGCTCA	AGCCTAGGGCTCTGCTCCAGATA
Slc2a1	GTCGGACCCTGCATCTCAT	CTGAACAGCTCGGCCACAA
Slc2a4	CTCCAACTGGACCTGTAACTTCATC	GCCTCTGGTTTCAGGCACTC
Suclg2	GTGTAAAGGAGTCCCAAGTCTATC	GTTGACGATCCCACCAAAGA
Hk2	TCGATGGCTCCGTCTACAAGAA	ACATCACAGTCGGGCACCAG
Pfkm	GGATATGATACCAGGGTCACTGTTC	AAAGTGCCATCACTGCTTCCA
Pkm	CGATCTGTGGAGATGCTGAAGG	GGCTGCACGGACATTCTTGA
Tpi	CCTGGCATGATCAAGGACTTAG	GGATAGGGCATGGTTCACTTT
Ldha	GACTTGGCCGAGAGCATAAT	GGAAGACATCCTCCTTGATTCC
Pdk4	TGAACCAGCACATCCTCATATT	CTTCGACTACTGCTACCACATC
Ppara	TGCTGAAGTACGGTGTGTATG	CTTTAGGAACTCTCGGGTGATG

Table S4. Composition of the BCAA-free DMEM medium.

Component	Concentration (mM)
Glycine	0.4000
L-Arginine hydrochloride	0.3981
L-Cystine 2HCl	0.2013
L-Glutamine	4.000
L-Histidine hydrochloride-H_2O	0.2000
L-Lysine hydrochloride	0.7978
L-Methionine	0.2013
L-Phenylalanine	0.4000
L-Serine	0.4000
L-Threonine	0.7983
L-Tryptophan	0.07843
L-Tyrosine disodium salt dihydrate	0.3985
Choline chloride	0.02857
D-Calcium pantothenate	0.008386
Folic Acid	0.009070
Niacinamide	0.03279
Pyridoxine hydrochloride	0.01942
Riboflavin	0.001064
Thiamine hydrochloride	0.01187
i-Inositol	0.04000
Calcium Chloride ($CaCl_2$)	1.802
Ferric Nitrate	0.0002480
Magnesium Sulfate ($MgSO_4$)	0.8139
Potassium Chloride (KCl)	5.333
Sodium Bicarbonate ($NaHCO_3$)	44.05
Sodium Chloride (NaCl)	110.3
Sodium Phosphate monobasic (NaH_2PO4-H_2O)	0.9058
D-Glucose (Dextrose)	25.00
Phenol Red	0.03985

Table S5. Composition of the BCAA-free substrate-limited DMEM medium for seahorse analysis.

Component	Concentration (mM)
Glycine	0.4000
L-Arginine hydrochloride	0.3981
L-Cystine 2HCl	0.2013
GlutaMAX	1.000
L-Histidine hydrochloride-H_2O	0.2000
L-Lysine hydrochloride	0.7978
L-Methionine	0.2013
L-Phenylalanine	0.4000
L-Serine	0.4000
L-Threonine	0.7983
L-Tryptophan	0.07843
L-Tyrosine disodium salt dihydrate	0.3985
Choline chloride	0.02857
D-Calcium pantothenate	0.008386
Folic Acid	0.009070
Niacinamide	0.03279
Pyridoxine hydrochloride	0.01942
Riboflavin	0.001064
Thiamine hydrochloride	0.01187
i-Inositol	0.04000
Calcium Chloride ($CaCl_2$)	1.802
Magnesium Sulfate ($MgSO_4$)	0.8139
Potassium Chloride (KCl)	5.333
Sodium Chloride (NaCl)	110.3
Sodium Phosphate monobasic (NaH_2PO4-H_2O)	0.9058
D-Glucose (Dextrose)	0.5000
Phenol Red	0.03985
Carnitine	0.5000

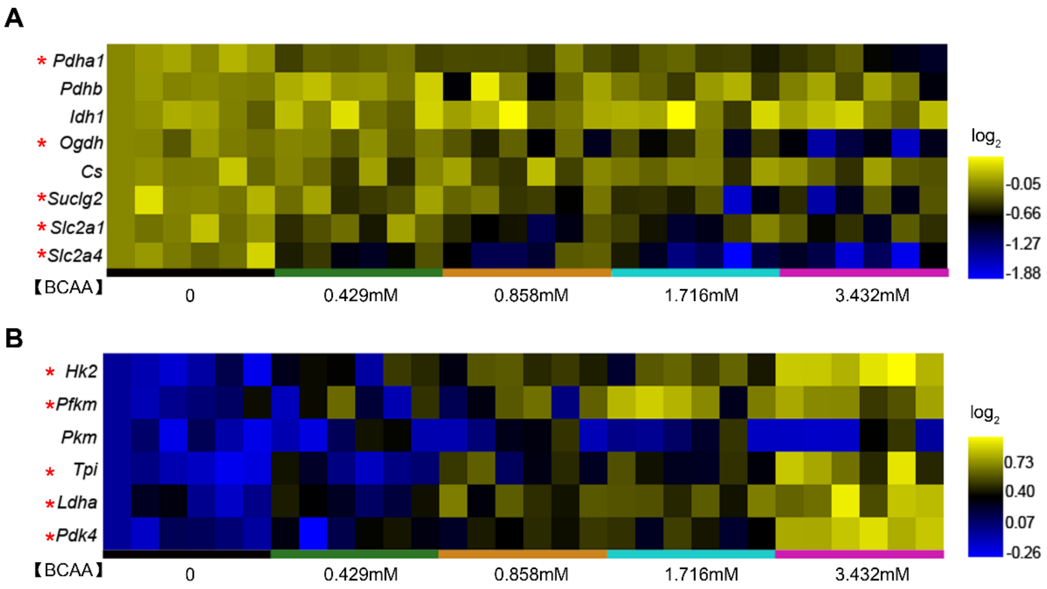

Figure S1. The effect of BCAA on mRNA levels of glucose oxidation and glycolysis-related enzymes in cardiac myocytes. Adult mouse cardiac myocytes were treated with increased concentrations of BCAA for 12 hours as indicated. (A) mRNA levels of enzymes catalyzing glucose oxidation were determined by RT-PCR and were shown by heat map. (n=6); (B) mRNA levels of enzymes catalyzing glycolysis were determined by RT-PCR and were shown by heat map. (n=6).

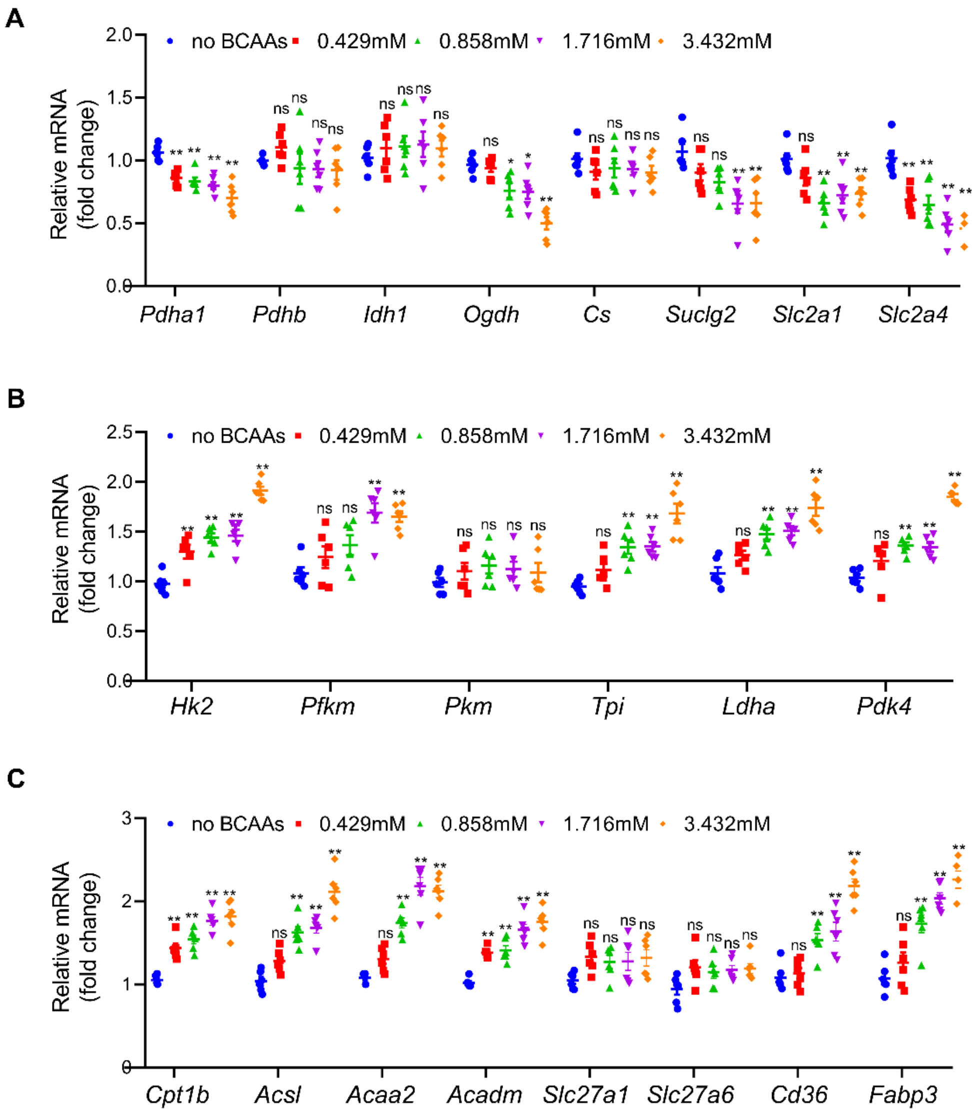

Figure S2. The effect of BCAA on mRNA levels of enzymes catalyzing glucose oxidation, glycolysis, and fatty acid oxidation (FAO) in cardiac myocytes. Adult mouse cardiac myocytes were treated with increased concentrations of BCAA for 12 hours as indicated. mRNA levels of enzymes catalyzing glucose oxidation (A), glycolysis (B) and FAO (C) were determined by RT-PCR (n=6). Data were analyzed by one-way ANOVA, followed by a Bonferroni post-hoc test. * $P<0.05$, ** $P<0.01$. All values are presented as mean ± SEM.

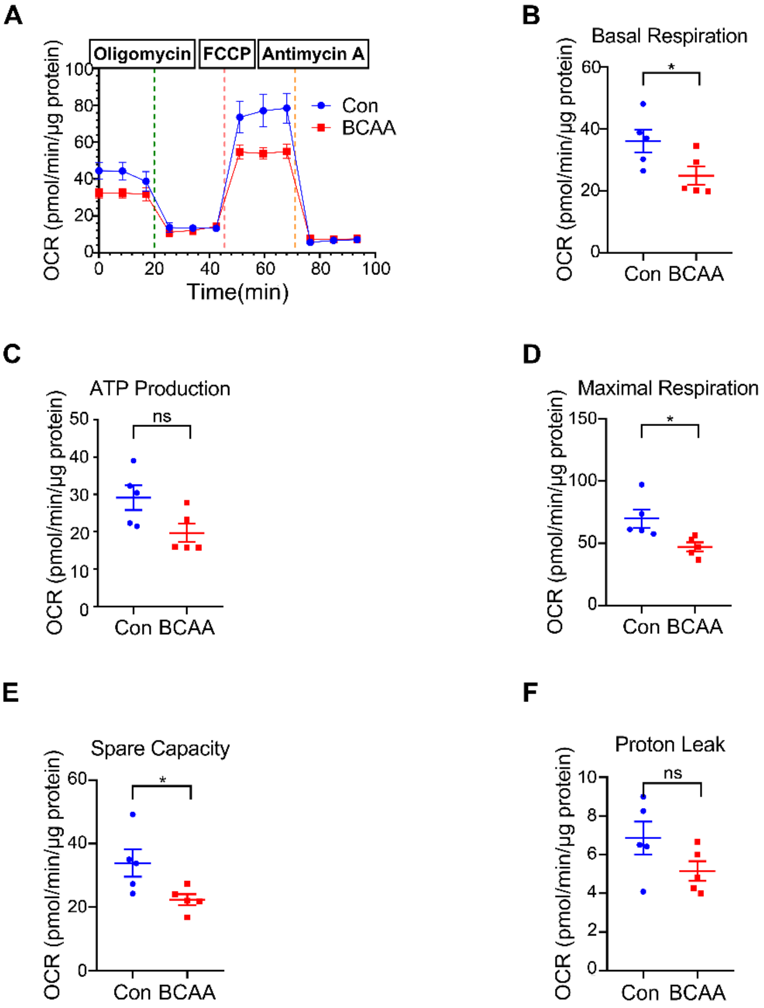

Figure S3. The effect of BCAA on glucose oxidation in cardiac myocytes. Adult mouse cardiac myocytes were isolated and incubated with non-BCAA (Con) or BCAA (3.432 mM) for 12 hours. Glucose oxidation was determined by seahorse analyzer (n=4-5). (A) OCR curve treated as mentioned above were determined. (B) Basal respiration (C) ATP production (D) maximal respiration (E) spare capacity and (F) proton leak were calculated according to instruction. Data were analyzed by Student's t test (two-tailed). * P<0.05. All values are presented as mean ± SEM.

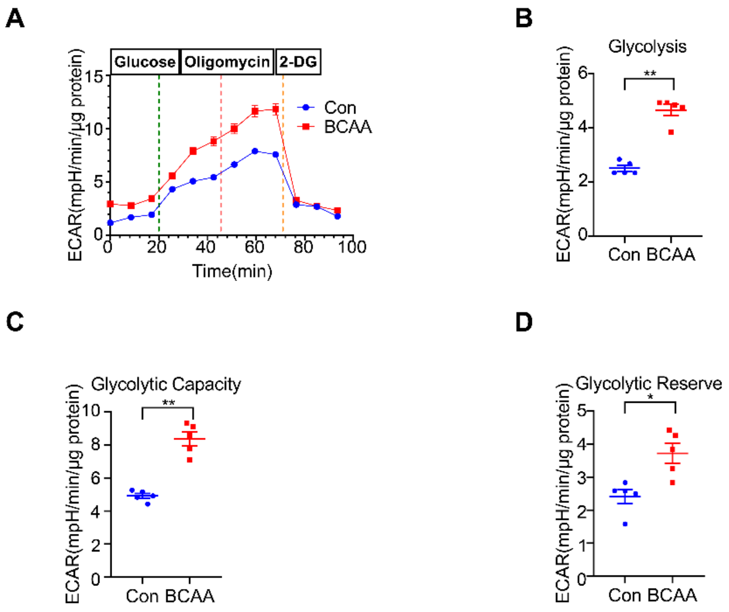

Figure S4. The effect of BCAA on glycolysis in cardiac myocytes. Adult mouse cardiac myocytes were isolated and incubated with non-BCAA (Con) or BCAA (3.432 mM) for 12 hours. Glycolysis was determined by seahorse analyzer (n=4-5). (A) ECAR curve treated as mentioned above were determined. (B) Glycolysis (C) Glycolytic capacity and (D) glycolytic reserve were calculated according to instruction. Data were analyzed by Student's t test (two-tailed). * $P<0.05$, ** $P<0.01$. All values are presented as mean ± SEM.

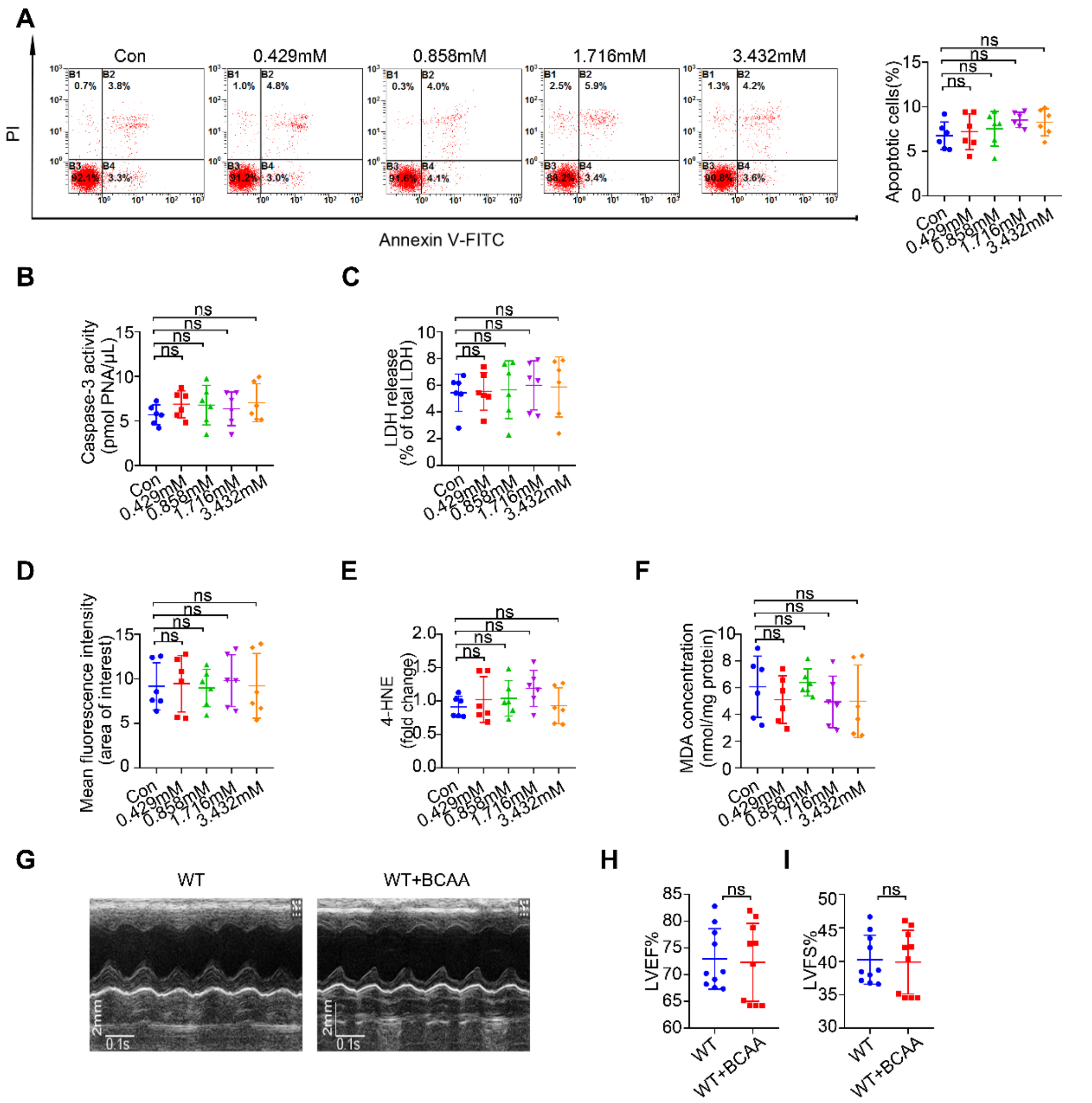

Figure S5. BCAA had no deleterious effect on cardiomyocyte survival, superoxide generation, lipid peroxidation and cardiac function under basal conditions. (A-F) NRVMs were isolated and treated with different concentrations of BCAA (0, 0.429 mM, 0.858 mM, 1.716 mM, 3.432 mM) for 12 h. (A) Apoptosis was analyzed by Annexin V-FITC flow cytometry (n=6). (B) Caspase 3 activity detection (n=6). (C) LDH release determination (n=6). (D) Superoxide generation was assessed by DHE staining (n=6). (E-F) 4-HNE and MDA levels were determined (n=6). (G) Representative M-Mode echocardiographic images. (H-I) Echocardiographic assessment of LV ejection fraction and LV fractional shortening (n=8-10). (A-F) Data were analyzed by one-way ANOVA, followed by a Bonferroni post-hoc test. (H-I) Data were analyzed by Student's t test (two-tailed). * P<0.05, ** P<0.01. All values are presented as mean ± SEM.

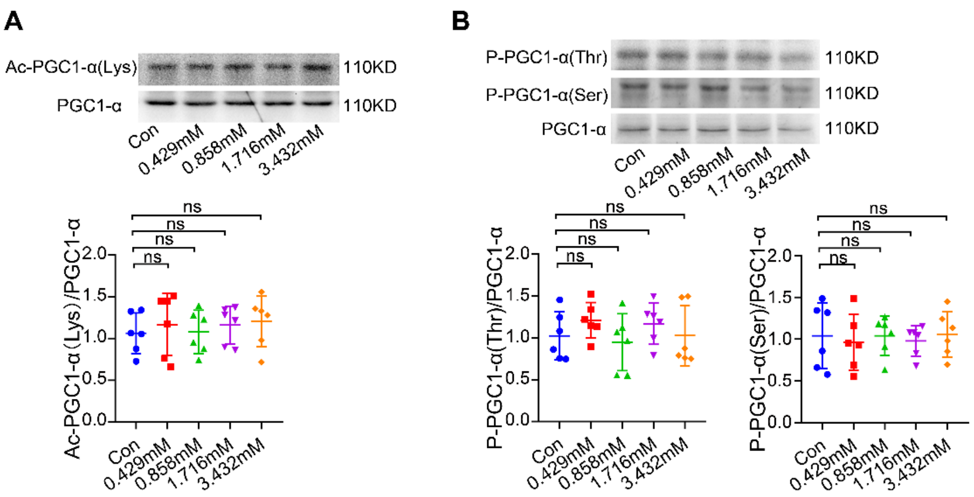

Figure S6. The effect of BCAA on the acetylation and phosphorylation levels of PGC-1α in NRVMs. (A-B) NRVMs were isolated and treated with different concentrations of BCAA (0, 0.429 mM, 0.858 mM, 1.716 mM, 3.432 mM) for 12 h, and the myocytes lysates were immunoprecipitated with anti-PGC1-α antibody overnight and blotted with anti-acetylated-lysine antibody (cell signaling technology, 9441S), anti-phospho-threonine antibody (abcam, ab9337) or anti-phospho-serine antibody (abcam, ab9332). (A) Expression of acetylated PGC-1α by western blotting (n=6). (B) Expression of phosphorylated PGC-1α by western blotting (n=6). Data were analyzed by one-way ANOVA, followed by a Bonferroni post-hoc test. All values are presented as mean ± SEM.

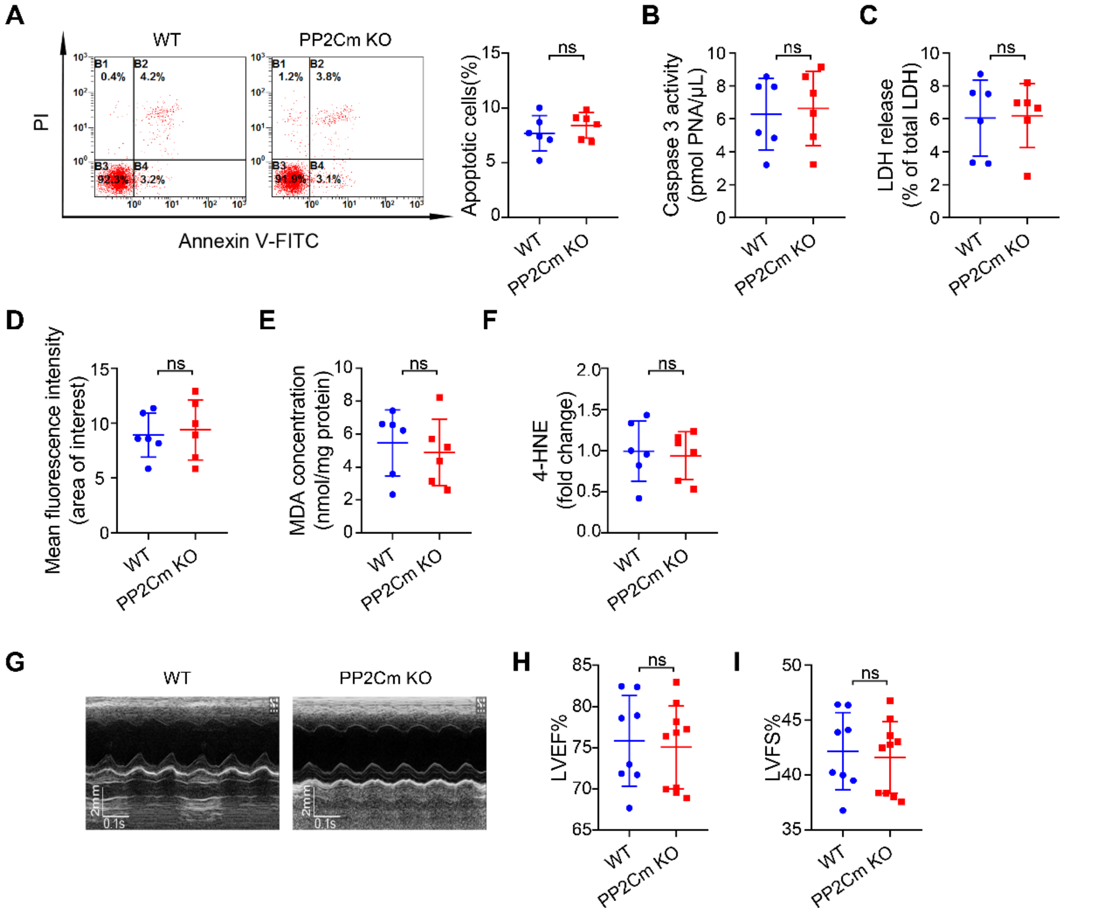

Figure S7. PP2Cm KO had no deleterious effect on cardiomyocyte survival, superoxide generation, lipid peroxidation and cardiac function under basal conditions. (A) Apoptosis was analyzed by Annexin V-FITC flow cytometry (n=6). (B) Caspase 3 activity detection (n=6). (C) LDH release (n=6). (D) Superoxide generation was assessed by DHE staining (n=6). (E-F) MDA and 4-HNE were determined (n=6). (G) Representative M-Mode echocardiographic images. (H-I) Echocardiographic assessment of LV ejection fraction and LV fractional shortening (n=8-10). Data were analyzed by Student's t test (two-tailed). * P<0.05, ** P<0.01. All values are presented as mean ± SEM.

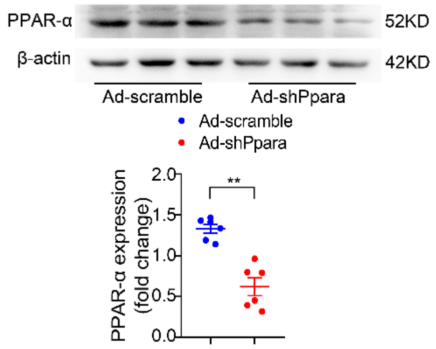

Figure S8. The effect of Ad-shPpara on cardiac PPAR-α Expression. Mice were received intra-myocardial injection of adenovirus carrying scramble (Ad-scramble) or shPpara (Ad-shPpara). 7 days post-injection, myocardial PPAR-α expression was determined by western blotting (n=6). Data were analyzed by Student's t test. ** P<0.01. All values are presented as mean ± SEM.
